# Naming speed during language production in younger and older adults: Examining the effects of sentence context

**DOI:** 10.1177/17470218241309602

**Published:** 2025-01-08

**Authors:** Naveen Hanif, Elizabeth Jefferies, Angela de Bruin

**Affiliations:** Department of Psychology, University of York, York, UK

**Keywords:** Language production, cognitive ageing, semantic control, sentence context

## Abstract

Word retrieval during speech production has been found to slow down with ageing. Usually, words are produced in sentence contexts. The current studies examined how different sentence contexts influence lexical retrieval in younger and older adults. We also examined the potential influence of semantic knowledge and control on sentence-context effects. Study 1 was completed by 48 younger and 48 older adults. They named pictures that were preceded by a matched context (which predicted that specific target word), a mismatched context (predicting another word), a neutral context (that did not predict one specific word), or no context. In comparison to the neutral context, both younger and older adults’ word production was faster in matched contexts, suggesting both age groups benefitted from sentence contexts facilitating the retrieval of predictable words. Neither age group was slowed down by the mismatched contexts (compared to the neutral contexts), suggesting these contexts did not create (sufficient) interference to hinder lexical retrieval. In Study 2, participants completed measures of semantic knowledge, verbal fluency, semantic control, and inhibition. Older adults showed larger semantic knowledge but poorer inhibition and (on some measures) semantic control than younger adults. However, none of these measures predicted the sentence context effects observed in Study 1. Together, this suggests older adults’ lexical retrieval can continue to benefit from sentence contexts predictive of upcoming words during language production.

## Introduction

The number of people aged 65 years and over is increasing rapidly (Office for National Statistics [ONS], 2021). Ageing has consequences for language production, including lexical retrieval. This is the process through which words stored in the mental lexicon are accessed for production (cf. Friedmann et al., 2013). Lexical retrieval difficulties are the most common type of memory difficulty experienced by older adults (e.g., Ossher et al., 2013). In particular, the efficiency (speed) with which words are retrieved from memory declines with age (Verhaegen & Poncelet, 2013). In the existing literature, lexical retrieval has most commonly been studied in isolation (e.g., during picture naming). However, in everyday conversation, language is often used to respond to other people, for example, when they ask you a question. The sentence context (e.g., the nature of the question asked by our conversational partner) might influence how quickly we can retrieve the words we need to respond to them. Across two studies, we therefore examined lexical retrieval efficiency in younger and older adults, in response to different types of sentence contexts. We also examined how cognitive factors (particularly those known to be affected by ageing) influenced the younger and older adults’ ability to respond to the different sentence contexts.

### Lexical retrieval in older adults

Picture-naming tasks often show that older adults (typically defined as aged 65 years or older) have more difficulty naming objects than younger adults (e.g., Barresi et al., 2000; Connor et al., 2004; Goral et al., 2007; MacKay et al., 2005). While accuracy can sometimes be preserved in older adults (Boudiaf et al., 2018; Ferré et al., 2020; Hoyau et al., 2017; Schmitter-Edgecombe et al., 2000), or only show age-group differences after the age of 70 (Wen & Dong, 2023), naming times appear more sensitive to age-related changes at a relatively earlier age (e.g., Ferré et al., 2020; Hoyau et al., 2017). For instance, slower naming compared to younger adults has been observed from the age of 50 years (e.g., Verhaegen & Poncelet, 2013). Indeed, while the vocabulary itself (i.e., the knowledge) remains intact or even increases with age (e.g., Hoffman, 2018), it is the (speed of) *retrieval* of words that appears impacted. This can affect low-frequency words in particular (e.g., Ferré et al., 2020).

These changes in lexical retrieval can be explained through several cognitive changes generally observed with ageing, including general age-related slowing (e.g., Salthouse, 1996) and difficulties suppressing goal-irrelevant information (e.g., Hasher & Zacks, 1988). Focusing on language specifically, the transmission deficit hypothesis explains these findings through weakened connections between representations at different levels within the ageing lexicon (Burke et al., 1991). While semantic information about words can remain intact, this account argues that weaker connections from the lexical to the phonological level can result in poorer lexical retrieval in older adults.

### Language in context

Daily-life lexical retrieval typically takes place in context, including sentences and interaction with other people. Studies examining connected speech in older and younger adults have shown mixed findings regarding age-related differences. Some studies report that older adults perform more poorly in connected speech tasks than younger adults do (e.g., older adults produce fewer words and make more word-choice errors, Heller & Dobbs, 1993). Other studies have reported similar performance in older and younger adults on several measures (e.g., in terms of the number of words produced and dysfluency during neutral picture descriptions; Castro & James, 2014) or show that older adults produce more words than younger adults during connected speech (e.g., Arbuckle et al., 2000; see the article by Kavé & Goral, 2017 for a review). Based on their review, Kavé and Goral (2017, p. 521) concluded that “*there is little evidence for significant word retrieval deficits in connected speech production in healthy aging*.” Furthermore, performance on picture-naming and connected speech tasks does not always correlate (e.g., Saling et al., 2012; Schmitter-Edgecombe et al., 2000). This might in part be related to the different measures used. Connected speech studies have used a range of variables, including number of words produced, lexical diversity (variety in the words used), word substitutions and circumlocutions (suggesting failed target-word retrieval), coherence, and dysfluency (e.g., Hoffman et al., 2018; Kavé & Goral, 2017). Picture-naming tasks, however, often measure *speed* of lexical retrieval (naming times) and/or accuracy. These measures might tap into different aspects of language production and thus make it difficult to directly compare retrieval in context (connected speech) versus in isolation (picture-naming times measured during picture naming might be most sensitive to detecting earlier and smaller changes in lexical retrieval; e.g., Verhaegen & Poncelet, 2013). Furthermore, some measures used in connected speech might partly assess *compensatory strategies* (Kavé & Goral, 2017), for example, in the form of circumlocutions that can mask retrieval difficulties (e.g., Nicholas et al., 1985). The various measures used across studies hinder a direct comparison, making it difficult to evaluate whether age-related effects on lexical retrieval and language production are truly influenced by the presence of context. In Study 1, we therefore assessed speed of picture (naming times) when pictures were presented in isolation and when they were preceded by a sentence context.

In addition to the *presence* of context, the *type* of context might influence lexical retrieval. Words are often retrieved faster when preceded by contexts with lexical-semantic information that is compatible with the target word. For example, following the question “*What is he woken up by every morning?*,” the picture of an *alarm clock* is named faster than after a semantically neutral question such as “*What did he hear yesterday?*” (Shao & Rommers, 2020). Upon hearing or seeing a word, activation of its semantic and/or lexical features can spread activation to neighbouring representations that share features or associations. This priming can facilitate production of related words, compared to unrelated words that do not share semantic features. This has also been linked to prediction, with listeners argued to predict upcoming words, potentially through pre-activation of specific lexical features that are likely to appear (e.g., Federmeier, 2007). In contexts where those predictions are accurate, this could facilitate the speaker’s own production. However, in daily-life speech and language, we often also encounter words that are not highly predictable (Luke & Christianson, 2016). Contexts that are not compatible with a target word (i.e., where the prediction is incorrect) can result in processing costs (e.g., Federmeier et al., 2003, but cf. Luke & Christianson, 2016). This could potentially slow down language production, although a recent study (Bannon et al., in press) suggested this might especially be the case when the unexpected words are not related to the predicted target word at all.

These semantic relationships can further modulate age-related differences in lexical retrieval as older adults have shown poorer semantic control, the mechanism through which intended representations (semantic knowledge) are retrieved from the semantic store while competing representations are suppressed (Hasher & Zacks, 1988; Jefferies, 2013). While older adults’ semantic knowledge is often reported to be comparable to, or even larger than, younger adults’ knowledge (e.g., Carrol, 2023; Hoffman, 2018; Hoffman et al., 2018; Kavé & Halamish, 2015; Kavé & Yafé, 2014; Verhaeghen, 2003), semantic control and inhibitory control diminish with age (e.g., Hasher & Zacks, 1988; Hoffman, 2018; Spieler et al., 1996). Weakened inhibitory control can create difficulties in suppressing responses and information irrelevant to the task at hand. The term “inhibitory control” is often used as a general term to refer to control over different types of information, both linguistic/verbal and non-linguistic/non-verbal. Semantic control specifically refers to control over irrelevant semantic representations (e.g., distractor words that are semantically related to the target while having to make a size judgement). Both semantic and inhibitory control have been found to predict some aspects of word production (Arbuckle & Gold, 1993; Hoffman et al., 2018; Yin & Peng, 2016; but see Higby et al., 2019). Age-related changes in semantic networks and control processes may thus have implications for lexical retrieval processes in everyday conversation.

Our Study 1, therefore, first examined lexical retrieval in older and younger adults in different sentence contexts that varied in their semantic relationships with the target word to be named. The preceding sentence was neutral (not semantically related to the target), predicted the target word (“matched,” e.g., “mountain” was predicted by “What did the alpinist climb?”), or predicted a different target (“mismatched,” e.g., “ladder” rather than the target “mountain” was predicted by “What did the construction worker climb?”). In Study 2, we examined whether lexical retrieval in these different contexts related to performance on other cognitive tasks, including semantic control and inhibition. Together, these studies allowed us to examine potential age-related changes in lexical retrieval during word production in sentence context, as well as the underlying mechanisms contributing to lexical retrieval in context. Specifically, considering the observed age-group differences in previous studies, our studies also aimed to better understand the potential impact of (age-related changes in) semantic knowledge and control on language production.

## Study 1

### Introduction

#### Sentence context

Previous literature has mostly assessed the role of sentence context in older adults through comprehension studies, with little work assessing production. Production has mostly been studied through priming paradigms manipulating the relationship between an individual prime word and target word (e.g., “doctor-nurse”). Both younger and older adults show faster naming when target words are preceded by a semantic prime than by a neutral prime (Balota et al., 1999; Faust et al., 2004), with some findings suggesting older adults may benefit even more from semantic priming than younger adults (see the article by Laver & Burke, 1993 for a review). The *comprehension* studies that have looked at word processing in sentence contexts have reached a range of conclusions. Some have found that both younger and older adults use sentence context to facilitate retrieval of upcoming words to the same extent (e.g., Kliegl et al., 2004, using eye tracking). Other findings suggest that older adults can utilise semantic cues to a *greater* degree than younger adults (e.g., Pichora-Fuller et al., 1995; Rayner et al., 2006), lending support to the hypothesis that older adults can utilise semantic context to help them to overcome age-related declines (see also the articles by Rayner et al., 2006; Speranza et al., 2000). However, other studies suggest that older adults do not use semantic information within sentential contexts as effectively as younger adults do. These findings are often shown through electroencephalogram (EEG) data examining N400 effects, a negative-going wave peaking approximately 400 ms after stimulus onset. This N400 effect is often reduced in amplitude in congruent sentence contexts, but such sentence context effect has been observed to be smaller or delayed for older than for younger adults (e.g., Federmeier et al., 2002; Wlotko et al., 2012).

Together, these findings suggest older adults can continue to use semantically congruent information during language processing, although it remains unknown whether they can benefit more or less than younger adults. These findings are based on studies looking at language comprehension (e.g., sentence processing), leaving it also largely unknown how congruent (“matched”) sentence contexts can influence language production in older adults.

Furthermore, the focus has been on facilitation stemming from semantically congruent information, but in contexts that are incongruent with a target word, older adults might experience more interference based on diminished inhibitory and/or semantic control. Studies looking at semantic interference at the individual word level (e.g., naming the picture of a *ball* while seeing the word *frisbee*) have shown both younger and older adults show slower naming in the presence of this distractor than when they see a neutral word. This semantic interference effect is often greater within older adults than in younger adults (e.g., Taylor & Burke, 2002), suggesting that older adults have more difficulty in inhibiting semantic distractors. However, this is not found across all studies, with Lorenz et al. (2019) showing a similar impact of semantic distractors on younger and older adults. Some event-related potential (ERP) studies, finally, have suggested that older adults might be influenced less by incongruent sentence contexts as a consequence of predicting upcoming words less or less successfully (e.g., Wlotko et al., 2012). However, these studies often compare unexpected, incongruent words to expected, congruent words without a neutral baseline. This makes it difficult to disentangle effects of (potentially facilitating) congruent contexts and (potentially interfering) incongruent contexts.

#### Rationale Study 1

The current literature thus has shown mixed effects regarding age-related differences in terms of lexical retrieval in context. It has furthermore focused on comprehension and, in the absence of a neutral baseline, often does not allow for a comparison between semantically congruent (matched) and incongruent (mismatched) contexts. Study 1 therefore examined the effects of both congruent (matched) and incongruent (mismatched) sentence contexts (relative to a neutral baseline) on lexical retrieval in younger and older adults. We used a picture-naming paradigm similar to that used by Shao and Rommers (2020), who showed faster naming in younger adults when naming a picture after a matching question that was related to that target word, compared to a neutral question. In addition, we also included a mismatching sentence context as well as a picture-naming task without context (see Table 1 for example stimuli).

**Table 1.** Examples of stimuli used in the picture-naming task in Study 1.

Target picture	Matched	Mismatched	Neutral	No context
Egg	*What did the chef crack?*	*What did the baby shake?*	*What did the father ask his son to bring?*	No question
Rattle	*What did the baby shake?*	*What did the chef crack?*	*What did the father ask his son to bring?*	No question

The first three columns relate to the three types of sentence contexts used in the study, including example sentences. The question always preceded the presentation of a target picture, which participants had to name. In the “no context” condition, participants just named the picture (without hearing a question beforehand).

In terms of our hypotheses, we first expected a “Match effect,” with faster picture naming after a matched than a neutral sentence. Based on previous literature, it was unclear if older adults’ Match effect would be similar to that of younger adults. Preserved or increased semantic knowledge in older adults (cf. Hoffman, 2018) might help both age groups equally to retrieve words in matched sentence contexts, or might help older adults even more than younger adults to compensate for slower lexical retrieval (larger Match effect). However, less-efficient use of semantic information (e.g., due to slower transmission between representations or less prediction forming, e.g., Dell, 1986; Federmeier et al., 2010) may result in a smaller or no Match effect for older adults.

Furthermore, we expected a “Mismatch effect” with slower naming in semantically mismatched contexts than in neutral contexts. In line with the inhibition deficit hypothesis (Hasher & Zacks, 1988) and decreased semantic control in older age (Hoffman, 2018), semantically mismatched information may be more likely to interrupt older adults’ retrieval of upcoming words (larger Mismatch effect). A similar Mismatch effect in younger and older adults would suggest that mechanisms used to inhibit competing words are not negatively affected by ageing. Finally, if older adults do not use semantic information to predict upcoming words to the same extent as younger adults, they might experience less interference (smaller Mismatch effect).

Finally, we aimed to examine whether context in general (producing picture names in response to a neutral question) can influence lexical retrieval, relative to no context (producing individual picture names). This was done in an attempt to overcome the issues faced previously when trying to compare word production across different tasks employing differing measures (Kavé & Goral, 2017). Age effects might be exacerbated in a relatively artificial task asking participants to produce individual words without the typical syntactic and lexical connections between words in context. Faster retrieval *within* a sentence context (compared to an isolated word) would suggest that those syntactic and lexical connections between words (as is common in daily-life speech) can aid lexical retrieval, even if the context does not provide clear semantic predictions. If older adults rely more heavily on contextual support to aid word retrieval, they may exhibit a larger context effect. On the other hand, listening and responding to another speaker may impose greater working memory demands than producing words without context. If older adults have more difficulty managing the working memory demands of producing words within conversation, they may be delayed by context (Kemtes & Kemper, 1997; Murphy et al., 2000).

### Methods

This study was pre-registered on the Open Science Framework: https://osf.io/8qexr/. The data for both studies are also available on that OSF page.

#### Participants

Ethical approval was obtained from the Department of Psychology at the University of York, and participants provided informed consent at the start of the study. The final sample included 96 native English-speaking monolinguals. Forty-eight older adults (aged 65–77 years old) were recruited through prolific.co (*n* = 34) and through our departmental database (*n* = 14). Forty-eight younger adults (aged 18–35 years) were recruited through SONA (the university’s internal participant recruitment system; *n* = 6) and Prolific (*n* = 42). Participants received monetary compensation, Amazon vouchers, or course credit for their participation. The groups of younger and older adults were matched on sex ratio, number of years of formal education received, and the number of participants within each age group who had completed at least an undergraduate degree (see Table 2).

**Table 2.** Details of participants included in Study 1.

	*N*	Age (in years)	Formal education (in years)	Graduate education	Sex		Handedness	
					Male	Female	Left	Right
Younger	48	24.25 (4.6) 18–35	16.5 (2.8)	29	25	23	10	38
Older	48	69 (4.1) 65–77	15.6 (3.4)	31	25	23	4	44

The details include age and formal education (mean number of years, standard deviations in parentheses, and age range below); graduate education (total number of participants who had completed at least an undergraduate degree); sex and handedness (total number of participants belonging to each category).

Participants were first asked to complete a series of checks, including testing their microphone and playing audio files. We checked these responses before inviting them to the full study. Three participants were not invited as they could not complete the pre-study checks; one participant did not respond to the invitation; and three participants completed the study but were not included as they did not follow the instructions or because their naming-task recordings were empty. Our final sample size of 96 participants (as pre-registered, and after exclusion) was based on a GPower analysis. It was not possible to retrieve previous effect sizes from the literature as comparable designs/tasks had not been used previously with younger and older adults. We therefore conducted a power analysis using a medium effect size (*f* = 0.25) for an interaction between age and sentence context. This suggested our sample size yielded over 95% power to detect a medium-sized effect.

All included participants furthermore confirmed meeting the following eligibility requirements: they did not use a hearing aid, had (corrected-to-)normal vision, were not colour blind, had not been using medication affecting their concentration in the past 3 months, did not have a language/reading disability, and had not been diagnosed with a neurodegenerative disease or cognitive impairment. Given that the study was conducted online, we were not able to use an assessment of cognitive functioning such as the Addenbrooke’s Cognitive Examination (ACE-III). In addition to asking participants to confirm each eligibility point, where possible, we also used existing screening criteria to only invite participants without a history of head injury, cognitive impairment, or dementia.

#### Design

Participants completed a picture-naming task with the within-participant independent variable Context. This had four levels (see Table 1 for examples): Matched, Mismatched, Neutral, or No Context. Age group was a between-subject variable. The dependent variable was picture-naming times (ms), defined as onset of naming relative to picture presentation.

#### Materials

All target pictures for the naming task were presented in greyscale and were sourced from the Multipic database (Duñabeitia et al., 2018) or from Google images. Pictures were preceded by a spoken question or presented without context. The questions were recorded by a female English speaker, reflecting natural speech as much as possible. They were pre-processed using Praat (Boersma & Weenink, 2022) to add 50 ms to the beginning and end of each recording and to scale all recordings to 60 dB. Background noise was also reduced using Audacity^®^ version 3.0.0.

We created 76 matched question-answer pairs, in which the question was strongly predictive of the upcoming picture (see Table 1). Each matched pair was combined with another matched pair to create a duo (e.g., in Table 1, “egg” and “rattle” form a duo). Duos were formed on the basis that the sentence formed a match with one target word but a mismatch with the other target in the duo. We created mismatch sentences in which the target word was unlikely to follow but not impossible, to avoid unrealistic scenarios that would never happen in real-life conversations. Each duo was also assigned a neutral question, which did not strongly prime a specific word.

Each participant named each picture four times: three times within context (once per matched, mismatched, and neutral question) and once without context. We ensured that participants only heard each question once so that they could not use previous exposure to predict a word. Using the example presented in Table 1, half of the participants named “egg” four times in the four conditions while the other half named “rattle” four times in the same contexts. Matched and mismatched contexts were therefore the same questions across participants. Neutral questions were matched to the matched/mismatched questions in terms of overall sentence length (number of words) and syllable length and frequency of the key words. The full list of stimuli, with further details about the sentence characteristics and matching, is provided in the online Supplementary Materials.

To make sure the stimuli functioned as intended (i.e., target words were most likely in matched contexts and least likely in mismatched contexts), we ran three pilot studies, as described in the following sections. The pilot studies were completed online with our initial set of stimuli. Participants were recruited through SONA, Qualtrics, and Prolific for all three pilots.

The pilot studies included 47 sets of initially prepared stimuli (comprising *n* = 47 each of matched, mismatched, and neutral questions). Changes to the stimuli were made based on the pilot responses. The final set of stimuli was also evaluated through a likeliness rating task in the main study. This confirmed that target words were most likely in matched contexts and least likely in mismatched contexts (see “Results” for an analysis of these ratings).

One pilot study was a short written-picture naming task and was completed by five older (*M* Age = 63.6 years, range = 61–68 years) and six younger adults (*M* Age *=* 18.7 years, range = 18–20 years) to make sure all pictures could be recognised and named easily. We replaced pictures where this was not the case. Two further pilot studies were conducted to examine suitability of the stimulus materials in the different contexts. In the first study, 21 older (*M* Age = 65.7 years, range = 60–75 years) and 20 younger adults (*M* Age = 19.85 years, range = 18–31 years) completed a cloze probability task and a likeliness rating task. In the cloze probability task, they viewed each question individually and were asked to generate their first three single-word answers in response. We computed cloze probabilities (i.e., the proportion of times the target word was given as part of that “top three”) through by-item means rather than by-participant means, and in the following sections, we report scores only including items that were kept in the same form in the actual experiment. Cloze probability was highest for the matched condition in both age groups (younger: *M* = 80.52%, *SD* = 24.22; older *M* = 84.56%, *SD* = 19.77). This confirmed the matched target responses were indeed good answers to the questions. In contrast, as we wanted, the target words were almost never given in the mismatched context (younger *M* = 2.38%, *SD* = 6.80; older *M* = 2.78%, *SD* = 9.15) and neutral context (younger *M* = 1.72%, *SD* = 5.51; older *M* = 1.48%, *SD* = 5.65).

Participants in the pilot also completed a likeliness ratings task. They viewed each question-answer pair and were asked to rate on a scale of 1–5 how *likely* the presented answer was to follow the preceding question. Ratings (only including items that were kept in similar form in the actual experiment) were highest for matched pairs, followed by neutral pairs, and were lowest for mismatched pairs (see Table 3). A 2 × 3 analysis of variance (ANOVA) confirmed that there was a significant effect of Condition (*F*(1.663, 84.831) = 199.591, *p* < .001), with a significant difference between Matched and Mismatched (*p* < .001), Matched and Neutral (*p* < .001), and between Mismatched and Neutral (*p* = .015) likeliness ratings in the expected direction. Older adults overall provided slightly higher ratings (*F*(1, 51) = 38.832, *p* < .001), which interacted with Condition (*F*(1.718, 87.615) = 18.964, *p* < .001). This reflected that while younger and older adults rated likeliness of matched pairs similarly, older adults’ ratings of neutral and mismatched pairs were slightly higher than the younger adults’ ratings. However, analyses by age group confirmed that likeliness ratings were highest for the matched and lowest for the mismatched question-answer pairs in each age group individually.

**Table 3.** Pilot 1 likeliness data: mean likeliness ratings for matched, neutral, and mismatched question-answer pairs obtained from younger and older adults.

	Younger adults	Older adults
Likeliness rating (1–5)		
*Matched*	4.89 (0.13)	4.88 (0.26)
*Neutral*	2.70 (0.87)	3.12 (0.83)
*Mismatched*	2.27 (0.79)	2.55 (0.91)

Note that contrary to the main experiment, the pilot used a 1–5 rating scale (1 = *very unlikely*, 5 = *very likely*).

Finally, we asked participants to indicate whether the scenario depicted in each question-answer pair was possible (i.e., whether it was something that could happen in real life, as we wanted the mismatched sentences to be unlikely but not impossible) and whether the question-answer pairs were grammatically correct. For both questions, the answer options were “yes” (can happen in real life/grammatical) or “no” (cannot happen in real life/not grammatical). Both plausibility and grammaticality judgements (inferred from the mean number of participants who agreed that the scenarios could happen in real life and that they were grammatically correct) were high for all question-answer pairs we included.

After making modifications to the stimuli based on the results from the first pilot studies, we then conducted another pilot study with another five younger (*M* Age = 22.8 years, range = 19–29 years) and older adults (*M* age = 63 years, range = 60–67 years). Scores for the modified stimuli again, in line with pilot described earlier, confirmed that the targets were most likely to follow matched questions and least likely to follow mismatched questions.

#### Procedure

The experiment was conducted using Gorilla.sc (Anwyl-Irvine et al., 2020). Participants first read the information sheet and provided informed consent. They then completed a background questionnaire (see “Participants”). Next, they completed a sound check to ensure that they could record audio files through their browser and to adjust their device’s volume so that they were able to clearly hear the sentences. For the naming task, participants were allocated to one of 12 experiment lists. Half of the participants named the Set 1 targets, and the other half, the Set 2 targets (see the online Supplementary Materials). Furthermore, half of the participants named the pictures with context first, while the other half named the pictures without context first. Participants named 38 target words four times each, once without context and once in each of the three question contexts (114 trials, with a break in the middle). The presentation order of the stimuli was pseudo-randomised in the context blocks so that the same word was not repeated twice in a row, and there were no more than three consecutive trials of the same type of context.

Participants first completed a picture familiarisation task in which they saw the target pictures and words, asking them to read the word aloud and use it during the task. This phase was included to make sure all participants recognised the pictures when naming them in the study. Given that pictures were repeated within the task across the four conditions, including a familiarisation phase ensured participants did not see the picture for the first time within the main task, which could have affected condition comparisons. In the naming tasks, participants were instructed to name the pictures as quickly and accurately as possible. Participants first saw three practice trials. In the Context blocks, participants first viewed a fixation cross (500 ms) followed by a blank screen while they heard a pre-recorded question. This was followed by another fixation cross (presented for 500 ms). Next, the article “the” was presented on the screen (500 ms), and then another fixation cross (300 ms) was presented before the picture was presented. The picture remained on screen for 2500 ms, regardless of when a response was given. In the No-Context block, participants viewed a single fixation cross of the mean duration of the sentence recordings in the Context blocks, plus the duration of the fixation crosses (total of 3853.8 ms). The rest of the trial was identical to the Context trials (article followed by picture).

We also assessed participants’ subjective experienced workload using the NASA Task Load Index (NASA-TLX, Hart & Staveland, 1988). This task was used to examine (potential) differences in younger and older adults’ experienced subjective demands (rated on a scale of 1 (very low) to 100 (very high)) during the naming task. This allowed us to assess potential age-group differences not just in terms of objective performance (i.e., RTs, reaction times) but also in terms of experienced workload, which might be higher for older than for younger adults. After each naming block, participants provided ratings evaluating how mentally demanding, physically demanding, and temporally demanding (pace of the block) they found the task, as well as their performance (how successful they felt in terms of following the task instructions), effort (how hard they had to work), and their frustration level. We also assessed “overall workload” by asking participants to complete the full NASA-TLX after finishing the full naming task. This again asked participants to complete the same ratings (listed above), but we now also asked participants which aspect (e.g., “effort” versus “mental demand,” asking this question for each combination of the six experiences) they found more important when describing the experienced workload. This allowed us to compute scores reflecting the participants’ experienced workload per part of the task, as well as an overall score that also took into consideration that different aspects of workload experiences vary in how important they are for individual participants.

Finally, participants completed a likeliness-rating task in which they rated on a scale of 1–7 how likely the target word was to follow each question, for all of the question-answer pairs they viewed during the naming study. The experiment lasted approximately 30 to 45 min in total.

#### Data analysis

##### Likeliness ratings

Likeliness ratings in the main study were examined using a 2 × 3 ANOVA with Age (younger, older) as a between-subject variable and Context (matched, mismatched, neutral) as a within-subject variable. Data from two older participants were excluded from the likeliness-ratings analyses. Due to a technical fault, one participant was unable to use the ratings scale to indicate a likeliness rating of greater than “3.” The other excluded participant provided a rating of “2” on all trials. Given that Mauchly’s test of sphericity (for this task and all others) indicated the assumption of sphericity was violated for the Context variable, Greenhouse-Geisser corrected values are reported.

##### Picture naming

An accurate response in the picture-naming task was either the intended target word or a closely related word (e.g., “painter” instead of “artist”). Other or no responses were scored as an inaccurate response. Picture naming accuracy was >75% for all participants (*M* older adults 96.04%, *SD* = 4.92; *M* younger adults 97.00%, *SD* = 3.37). As pre-registered, because accuracy was close to ceiling, it was not analysed further. Naming RTs were determined using Checkvocal (Protopapas, 2007). RTs < 300 ms or more than 2.5 *SD* above or below the mean per participant and per condition were removed, using the trimr package (Grange, 2015; removing 2.78% of correct responses). With the exception of the data for the older adults in the Mismatch condition, Shapiro-Wilk tests confirmed the data were normally distributed (*p*s > .25).

RTs were analysed in SPSS using a 2 × 4 ANOVA to determine whether there was a main effect of Age, Context (i.e., matched, mismatched, neutral, without context), or an interaction between the two. If an effect of context was found, a pairwise comparison (Bonferroni corrected) was used to establish where the effect resided within the four levels. Given that we were specifically interested in the effect of each specific type of context and because we wanted to examine them while also accounting for age-related slowing, we then also computed the Match, Mismatch, and Context effects based on z-scored RTs for each participant (z-scored separately per age group). The Match effect was the RT difference between the matched and neutral questions; the Mismatch effect was the difference between mismatched and neutral questions; and the Context effect was the difference between neutral questions and naming without context. In addition to a one-way ANOVA per effect (using a Bonferroni adjusted significance threshold of *p* = .016 to account for the three comparisons), we computed a Bayesian ANOVA using JASP version 0.17.3 (JASP Team, 2024), which examined evidence for/against an age-group difference on these contextual effects. For each contrast effect (Matched, Mismatched, Context), we compared a model including an age effect (between-groups difference) to a null model (no age-group difference). We report these results in the form of “BF_01_,” showing the evidence for the null hypotheses (no age group difference) over the alternative hypotheses (significant age group differences). Values below 1 indicate evidence for an age-group difference; values above 1 indicate evidence for no age-group difference.

Finally, we estimated the internal consistency of the Match, Mismatch, and Context effects using a permutation-based split-half approach (Parsons, 2021) with 5,000 random splits to check for within-subject variations in these effects.

Further exploratory analyses used linear mixed-effect analyses to examine the context effects while considering both participants and stimuli within one analysis. We also examined the potential role of a specific target word’s likeliness scores as provided by the participants after completing the naming task. Finally, we examined the potential influence of lexical frequency, considering that older adults have shown increased difficulty retrieving low-frequency words. These analyses were conducted using *R* (4.4.1; lme4 package version 1.1.35) and started with the maximal random-effect structure including all within-participant and within-item slopes (following the article by Barr et al., 2013). Where analyses did not converge, we removed slopes explaining the lowest amount of variance until convergence was reached. Two-level categorical variables were contrast-coded (Age group: younger adults = −0.5; older adults = 0.5). Simple coding was used for the four-level categorical variable Context. “Neutral” was used as the reference level to compare the other three levels to that baseline (contrary to dummy coding, the intercept corresponds to the mean of all cell means). The continuous variables’ item frequency and item likeliness rating were z-scored.

##### Experienced workload (NASA-TLX)

Overall workload effects from the NASA-TLX were calculated by counting how often participants chose each experience as most important between two comparison options (e.g., how often they said they found “frustration” the most important compared to another experience in post-test evaluation). The raw score for each experience was computed and multiplied by the number of times it was chosen as most important. All weighted experiences were summed up and divided by the total number of comparisons participants had to choose from to compute the overall NASA score. We also calculated participants’ mean workload score after each block (by calculating the average of their ratings on each Likert-type scale, without weighing categories). An ANOVA was conducted to determine whether there was a difference in *overall* workload experience between older and younger adults. A mixed ANOVA established whether there was a significant effect of Context (ratings provided after No Context, the first half of Context, the second half of Context, and for the overall task) and age group on experienced workload.

### Results

#### Likeliness ratings

Target words in the matched Context were rated as most likely, and targets in the mismatched Context were rated as least likely (see Table 4, *F*(1.729, 159.107) = 2327.697, *p* < .001, η_p_^2^ = .962). Pairwise comparisons showed significant differences between all Context combinations (*p* < .001). There was no main effect of Age (*F*(1, 92) = 2.905, *p* = .092, η_p_^2^ = .031), suggesting that overall ratings were similar for older and younger adults. However, there was a significant interaction between Age and Context (*F*(1.729, 159.107) = 4.155, *p* = .022, η_p_^2^ = .043). While ratings of the matched and mismatched sentences were similar for both age groups (Matched: *p* = .718; Mismatched: *p* = .225), neutral targets were rated slightly more likely by younger than older adults (*p* = .025, see Table 4). Crucially, however, each age group showed a significant difference in likeliness ratings between all three question contexts (younger adults: *F*(1.656, 77.820) = 1029.568, *p* < .001, η_p_^2^ = .956; older adults: *F*(1.743, 78.441) = 1329.523, *p* < .001, η_p_^2^ = .967, with all pairwise comparisons *p* < .001 in both age groups). Thus, for both age groups, as intended, matched targets were most likely, followed by neutral and mismatched targets.

**Table 4.** Likeliness ratings obtained from participants completing the full study.

	Younger adults	Older adults
**Likeliness rating**		
*Matched*	6.69 (0.30)	6.72 (0.28)
*Neutral*	3.63 (0.64)	3.30 (0.73)
*Mismatched*	3.02 (0.56)	2.89 (0.53)

Note that contrary to the pilot, the main study used a 1–7 rating scale (1 = *very unlikely*, 7 = *very likely*).

#### Picture naming

**Figure 1.**
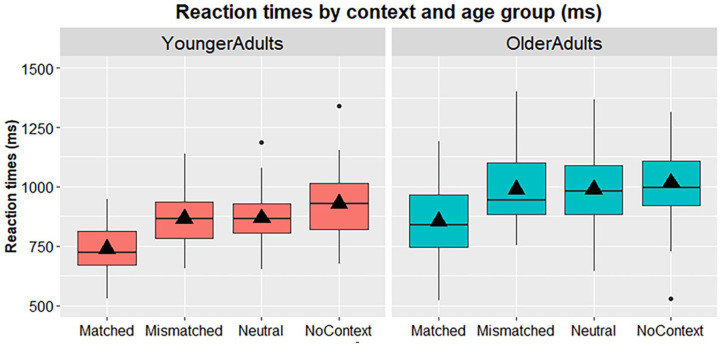
Box plots displaying untransformed mean RTs (ms) by age group (left panel: younger adults, right panel: older adults) and naming context. Box plot height denotes interquartile range; vertical lines below plots denote 25th percentile, while lines above denote 75th percentile. Black horizontal lines denote the median, triangles denote the mean. Black dots represent outliers.

We started the picture-naming analysis with the untransformed RTs. As expected, there was a significant main effect of Age group on RTs, with older adults taking significantly longer time to name pictures than younger adults (younger *M* = 848.70 ms, *SD* = 100.76; older *M* = 960.19, *SD* = 135.03; *F*(1, 94) = 21.358, *p* < .001, η_p_^2^ = .185). There was also a main effect of Context on picture-naming times (*F*(1.525, 143.337) = 110.600, *p* < .001, η_p_^2^ = .541). RTs were fastest in the matched trials, followed by the mismatched and neutral trials, and were slowest in the no-context trials (see Table 5, Figure 1). Pairwise comparisons showed that there were significant differences between RTs in all naming contexts (*p*s < .001 matched versus mismatched, neutral, and no-context; *p* = .005 for mismatched versus no-context; *p* = .008 for neutral versus no-context), apart from trials in the neutral and mismatched contexts (*p* > .999). This showed that matched contexts facilitated RTs compared to neutral contexts (Match effect) and that context facilitated RTs in comparison to naming without context. However, no Mismatch effect was observed, suggesting the mismatched context did not negatively affect production. Importantly, there was no significant interaction between Age group and Context (*F*(1.525, 143.337) = 1.266, *p* = .279, η_p_^2^ = .013), suggesting that the context effects did not differ between the younger and older adults.

**Table 5.** Mean picture naming times (and standard deviations) per Age group and Context.

Context	Younger adults	Older adults
*Without context*	927.95 (141.75)	1016.28 (164.47)
*Matched*	738.31 (102.86)	852.66 (145.03)
*Neutral*	867.25 (106.57)	988.50 (139.05)
*Mismatched*	864.16 (105.66)	988.36 (150.16)

##### Contrast Analyses for Match, Mismatch, and Context effects

**Figure 2.**
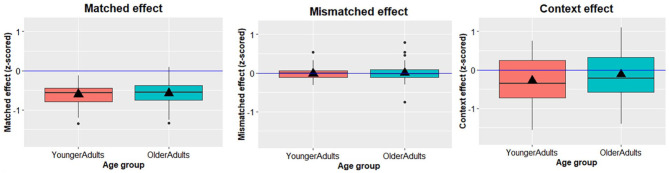
Box plots showing the facilitatory effects of matched contexts (left), no Mismatch effect in either group (middle), and facilitatory effect of (neutral) contex relative to naming without context (right) in both younger and older adults. Box plot height denotes interquartile range, vertical lines below plots denote 25th percentile, while lines above denote 75th percentile. Black horizontal lines denote the median, and triangles denote the mean. Black dots represent potential outliers.

As the previous analysis showed that there was a significant effect of age group on RTs, we z-scored the data to account for age-related slowing. Then, for each participant, we computed their Match effect (match versus neutral RTs), Mismatch effect (mismatch versus neutral), and Context effect (neutral context versus no-context; see Figure 2).

Starting with the Match effect, both the one-way ANOVA (*F*(1, 94) = 0.233, *p* = .630, η_p_^2^ = 0.002) and the Bayesian analysis (BF_01_ = 4.20, error = 0.02%) suggested the Match effect did not differ between age groups. The (Spearman-Brown corrected) split-half internal consistency of the Match effect was *r*_SB_ = 0.60, 95% CI [0.46, 0.72], indicating moderate internal consistency of this effect within participants.

Similarly, both analyses suggested the Mismatch effect did not differ between age groups either (one-way ANOVA: *F*(1, 94) = 0.116, *p* = .734, η_p_^2^ = 0.001; BF_01_ = 4.43, error = 0.02%). The (Spearman-Brown corrected) split-half internal consistency of the mismatch effect was *r*_SB_ = 0.19, 95% CI [−0.10, 0.43], suggesting low internal consistency of this effect within participants.

Finally, the one-way ANOVA again showed no age-group difference in the Context effect (*F*(1, 94) = 1.954, *p* = .165, η_p_^2^ = 0.020), although the Bayesian analysis only provided weak support for this null hypothesis (BF_01_ = 1.97, error = 0.02%). The (Spearman-Brown corrected) split-half internal consistency of the context effect was *r*_SB_ = 0.90, 95% CI [0.86, 0.93], suggesting high internal consistency of this effect within participants. While there is good internal reliability *within participants*, Figure 2 also shows high variability in the context effect across participants. Some participants exhibited context-related facilitation while others exhibited a context-related cost. We therefore conducted further exploratory analyses to examine if order (naming in context or without context first) could explain this variability. To that end, we computed a 2 × 2 ANOVA, with the Context effect as the dependent variable, and Age and Naming order (completing context or no-context part first) as independent variables. There was a significant main effect of naming order (*F*(1, 92) = 176.538, *p* < .001, η_p_^2^ = .657). Both younger and older adults showed a facilitatory effect of context only when the No-Context part was completed first (“context second group”: *M* younger = −0.745, *SD* = 0.382; *M* older = −0.603, *SD* = 0.295) but not when the Context part was completed first (“context first group”: *M* younger = 0.172, *SD* = 0.348; *M* older = 0.365, *SD* = 0.360). The effect of age was now significant (*F*(1, 92) = 5.584, *p* = .020, η_p_^2^ = .057), suggesting the context effect was slightly smaller for older adults, but this did not interact with naming order (*F*(1, 92) = 0.131, *p* = .718, η_p_^2^ = .001).

##### Exploratory analyses (pre-registered mixed-effect analyses exploring stimulus characteristics)

The analyses conducted so far used by-participant means. Given that language production is also influenced by the items used, we also ran a linear mixed-effect analysis. We report the analysis using untransformed RTs but also ran them using z-scored RTs, which showed the same findings (apart from the main effect of age group, as intended). The first mixed-effect analysis examined effects of Context and Age group, similar to the ANOVAs reported earlier, and converged with participant and item intercepts but no slopes. The Context findings were the same, with a Match effect (β = −134.128, *SE* = 4.103, *t* = −32.691, *p* < .001), no Mismatch effect (β = −2.836, *SE* = 4.127, *t* = −0.687, *p* = .492), and a Context effect (β = 42.732, *SE* = 4.118, *t* = 10.376, *p* < .001). The Match and Mismatch effects did not differ between age groups (Match × Age: β = −6.510, *SE* = 8.205, *t* = −0.793, *p* = .428; Mismatch × Age: β = 1.321, *SE* = 8.253, *t* = 0.160, *p* = .873). The Context effect was significantly smaller for older adults (β = −30.864, *SE* = 8.236, *t* = −3.747, *p* < .001).

Contrary to our predictions, there was no significant Mismatch effect. Likeliness ratings in the Mismatch condition were significantly lower than likeliness ratings in the other conditions, but within each condition, our stimuli varied in their likeliness. We therefore also conducted an exploratory analysis to examine whether there was a direct relationship between a participant’s likeliness rating for a given item and their RTs in response to that item. Across all conditions, higher likeliness ratings were associated with faster responses (β = −54.485, *SE* = 1.692, *t* = −32.198, *p* < .001). This was also the case when just considering the Mismatch trials (β = −12.514, *SE* = 3.755, *t* = −3.333, *p* < .001). None of the analyses, however, showed an interaction between likeliness and age group (all *ps* > .15).

A final exploratory analysis examined a potential role of word frequency, considering older adults have been found to have greater difficulty retrieving low-frequency words. No effects of frequency were observed on overall RTs (β = −5.249, *SE* = 6.743, *t* = −0.778, *p* = .439), and importantly, frequency did not interact with age group (β = 0.294, *SE* = 2.940, *t* = 0.100, *p* = .920). Frequency did not interact with Context either (all *p*s > .25).

#### Experienced workload (NASA-TLX)

Subjective workload experiences showed no main effect of Age (*F*(1, 94) = 0.052, *p* = . 820, η_p_^2^ = .001), suggesting that younger and older adults experienced workload similarly (see Table 6). Furthermore, there was no significant effect of Context (*F*(2.521, 236.968) = 2.378, *p* = .081, η_p_^2^ = .025), suggesting that workload did not differ as a result of naming with or without context or throughout the context blocks. This suggests that workload was consistent throughout the naming task. There was also no significant interaction between Context and Age (*F*(2.521, 236.968) = 0.224, *p* = .848, η_p_^2^ = .002). A one-way ANOVA also showed that overall workload (weighted for the importance of each experience per participant) did not differ between younger and older adults either (*F*(1, 94) = 0.055, *p* = .815, η_p_^2^ = .001).

**Table 6.** NASA-TLX workload scores.

	Younger adults	Older adults
**NASA**		
Single	24.59 (14.65)	24.91 (14.61)
Context 1	27.29 (13.63)	26.70 (15.60)
Context 2	26.30 (15.77)	25.23 (15.69)
After naming	25.02 (16.42)	23.70 (18.77)
**Overall NASA**	29.44 (17.92)	28.48 (21.91)

The first four rows show the means and standard deviations of older and younger adults’ NASA workload ratings after each naming block. “Overall NASA” reflects the weighted after-naming score for each age group.

### Discussion

Study 1 examined the influence of different sentence contexts on word production in healthy younger and older adults. We compared matched contexts that predicted upcoming words, neutral contexts that did not predict a specific word, and mismatched contexts that predicted alternative words, as well as naming words without context. The likeliness ratings confirmed that targets were indeed most likely to follow questions in the matched contexts and least likely to follow questions in the mismatched contexts. As expected, older adults were slower overall in the naming task than younger adults. Participants in both age groups showed a (similar) significant Match effect, reflecting faster naming in the matched than neutral contexts. No Mismatch effect (difference between mismatch and neutral contexts) was found in either age group. Some effect of context was observed in both age groups, although this was only present when participants had to name pictures without context first.

#### Facilitatory effects of semantically matching contexts

Similar to Shao and Rommers’ (2020) study with younger adults, our data showed that target-word production was faster after matched than after neutral sentences. The Match effect is likely related to semantic priming effects, wherein words in the context prime related words, making their retrieval faster. Listeners might furthermore be predicting upcoming words, and when these predictions are in line with the required response, production might be facilitated. This facilitation was found for both younger and healthy older adults to a similar degree. Previous research assessing context effects has mostly focused on comprehension and has returned mixed findings when comparing older and younger adults. Some of those studies have suggested similar benefits for older and younger adults (e.g., Kliegl et al., 2004) while others have suggested older adults benefit more (e.g., Pichora-Fuller et al., 1995) or less (e.g., Wlotko et al., 2012) from semantically related context than younger adults. However, many of these studies compared matched to incongruent or mismatched conditions, rather than to a neutral baseline. This often makes it difficult to attribute any context effects and age group similarities or differences to benefits associated with *matched* semantic content specifically. Our study shows that older adults can indeed benefit from semantic information matching upcoming target-word production. In this specific case, they did not benefit more than younger adults (unlike, e.g., Pichora-Fuller et al., 1995). However, such additional benefits for older adults might only arise when processing demands are high (e.g., when the task is presented in background noise, as done in the study by Pichora-Fuller et al., 1995). Furthermore, more compensatory effects (exhibiting a larger Match effect in older adults) might be more likely to arise in low-frequency target words with poorer lexical-phonological connections (James & MacKay, 2001). In contrast, our study used relatively high-frequency words, a familiarisation phase, and each word was produced multiple times to ensure the same words were used in all conditions. As a result, older adults may not have had to rely more heavily on semantic context than younger adults. However, the benefits observed are likely to require older adults to have intact semantic knowledge to facilitate priming of related words. Study 2 therefore first examined semantic knowledge in both younger and older adults, to test the hypothesis that older adults indeed continue to benefit from a semantic knowledge and vocabulary size at least comparable to that of younger adults. We furthermore examined whether individual differences in the size of context effects during language production can be explained by individual differences in semantic knowledge.

#### Mismatched sentence contexts

Picture-word interference paradigms without context have shown that distractor words semantically related to target pictures (e.g., target: ball, distractor word: frisbee) often slow down target word retrieval compared to unrelated distractors (e.g., hammer) in both younger and older adults (Taylor & Burke, 2002). This suggests that competing active semantic information can interfere with the production of intended words. However, in our study, mismatched sentence contexts did not influence target-word production times. Although we expected a Mismatch effect with slower responses when the target word was unexpected, these findings align with a previous comprehension study using comparable sentences (Haigh et al., 2022), where no Mismatch effects were found either. Similarly, Luke and Christianson (2016) showed no negative impact of unpredictable information in an eye-tracking study. Previous research (Brothers & Kuperberg, 2021) has suggested people engage less in predicting upcoming words when the context is uninformative. However, considering the observed Match effect, it is unlikely that the absent Mismatch effect in our study is due to the context’s overall low informative level resulting in people stopping predicting upcoming information entirely.

While our mismatched targets were *unlikely* answers to the preceding question, they were not *impossible*. Indeed, while likeliness ratings differed significantly between mismatched and neutral sentences, the differences were small. This is likely related to the mismatch trials not being unexpected enough, although the difference can also be increased by making the neutral trials more neutral/predictable. Previous comprehension studies have found the largest processing costs when sentence endings are semantic anomalies (e.g., Federmeier & Kutas, 1999). The mismatched contexts in the current study may not have increased competition between word candidates sufficiently to incur a cost, in particular, when comparing these effects to a neutral baseline (as opposed to a comparison to expected, matched contexts). Recent research also assessing production indeed showed only a small cost when unpredictable words were used that were closely related to the expected target (e.g., “skull” instead of “brain”) compared to a larger cost when the unpredictable word was not related to the expected target (Bannon et al., in press). Our exploratory analyses further suggested that, also within the mismatch context, responses were slower for target words that were less likely to occur in the sentence context. This suggests that a selection of impossible rather than unlikely target endings would perhaps be more likely to show significant interference effects. Variability between items in their (un)likeliness also likely contributes to the low split-half reliability observed for the Mismatch effect. However, age group did not interact with the effect of likeliness in the continuous rating analyses either, suggesting the older adults in this study were truly not affected more by target-word likeliness.

#### Neutral sentence contexts and ageing

Picture naming was faster following neutral contexts than after no context, suggesting sentence context facilitated production. Furthermore, this context facilitation appeared to be similar for both age groups, although some analyses suggested the effect was slightly smaller for older adults. Pictures were repeated throughout the task, and participants were exposed to them beforehand through a picture-familiarisation phase. It is possible that this facilitated older adults’ overall retrieval (compared to not having seen the picture or word beforehand) and that older adults benefit more from context when words are harder to retrieve (e.g., without previous exposure or when using lower-frequency words). Older and younger adults also experienced the workload in No-Context and Context conditions comparably, suggesting there was no age-group difference in perceived effort involved in naming with or without context.

However, follow-up analyses suggested the facilitatory effect of context in terms of naming times might not be entirely driven by effects of context as such. While the order of naming with or without context first was counterbalanced across participants, only participants who completed the naming task without context first showed faster naming within context. Given that pictures were repeated, it is possible that participants benefitted from naming those pictures without context first and therefore showed faster naming times in the sentence contexts. This could be because previous naming primed the lexical form (allowing for faster retrieval in the context task when completed second) and/or because participants expected the same pictures to appear again. A true effect of context facilitation on production should occur even if the context part is completed first. However, context effects varied among participants, and it is possible that other differences (e.g., individual differences in terms of working memory) contributed to the variability observed in context effects. This is assessed in more detail in Study 2.

## Study 2

### Introduction

In Study 2, we aimed to further examine the potential mechanisms underlying the context effects. Specifically, we studied how age groups differed in terms of cognitive and language variables (such as semantic knowledge and control, inhibition, and working memory) and assessed how these variables contributed to the context effects observed in Study 1. Note that this study was pre-registered before completing Study 1, and the hypotheses, therefore, do not take into consideration the Study 1 findings (e.g., the absence of a Mismatch effect). However, Study 2 focused mostly on trying to explain *individual differences* in these context effects, which were indeed observed in Study 1, despite the absence of group-level differences.

#### Semantic knowledge

As introduced in Study 1, semantic knowledge has been found to increase with age (e.g., Carrol, 2023; Hoffman, 2018; Hoffman et al., 2018; Kavé & Halamish, 2015; Kavé & Yafé, 2014; Verhaeghen, 2003). For example, in a synonym-selection task, Hoffman (2018) and Hoffman et al. (2018) asked younger and older adults to select the synonyms of probe words (e.g., “*which means the same as bombastic?*” answer: *pompous*, other answer options: *destructive, anxious*, *bickering*). In both studies, older adults provided significantly more correct answers than younger adults. Larger semantic knowledge scores might also relate to lexical retrieval. On the one hand, faster language production has been observed for participants with a larger vocabulary (Shao et al., 2014), suggesting greater semantic knowledge is associated with faster word retrieval. In older adults, their larger vocabulary may act as a compensatory defence against age-related lexical access difficulties (Juncos-Rabadán et al., 2010). On the other hand, having access to more words could create additional interference and disrupt language production (Ramscar et al., 2014). For example, Hoffman and colleagues (2018) found a negative relationship between semantic knowledge and coherence in connected speech.

The size of individuals’ semantic knowledge stores might particularly relate to the Match effect measured in Study 1. The Match effect is expected to depend on participants having access to semantic knowledge and connections, which allow for semantic priming and predictions about upcoming, semantically related words. In Study 2, we therefore used a synonym judgement task to assess semantic knowledge in the younger and older adults tested in Study 1, as well as a potential relationship between semantic knowledge and context effects (in particular, the Match effect). As discussed earlier, such a relationship could go in two directions. Greater semantic knowledge and a larger vocabulary inventory could facilitate word retrieval in matched contexts if they increase priming and allow participants to more easily predict upcoming words. Alternatively, having access to more semantic knowledge can increase interference and disrupt production (Ramscar et al., 2014). This could then result in smaller Match and potentially larger Mismatch effects.

#### Fluency

We also assessed semantic knowledge and lexical retrieval through verbal fluency tasks, which require participants to produce as many words as possible belonging to a specific category (semantic fluency) or beginning with a specific letter (letter fluency), within a specified time. Fluency has been linked to both lexical retrieval efficiency and semantic knowledge. Counter-intuitively, older adults often perform more poorly than younger adults on semantic fluency tasks while age-group differences tend to be smaller or absent on letter fluency tasks (e.g., Gordon et al., 2018; Kavé & Knafo-Noam, 2015). Here, we were predominantly interested in verbal fluency in general, given its links to both lexical retrieval efficiency and semantic knowledge across semantic and letter tasks (cf. Gordon et al., 2018). Verbal fluency has been associated with language production across younger and older adults (Higby et al., 2019). Greater semantic knowledge and lexical retrieval efficiency, as measured through verbal fluency scores, could help participants to benefit more from semantic connections in matched sentence contexts. We therefore examined whether verbal fluency (across letter and semantic fluency tasks) influenced the Match effect.

#### Semantic control

As discussed in the general Introduction, older adults have also shown declines in inhibition and semantic control (e.g., Hasher & Zacks, 1988; Hoffman, 2018). These control mechanisms are believed to relate to language production, where speakers need to inhibit competitors in favour of producing required words (e.g., Faroqi-Shah et al., 2014). This could be particularly pertinent in scenarios wherein speakers are required to produce unexpected words (i.e., the mismatched sentences in Study 1). Hoffman (2018) used a global and a feature semantic association task to assess semantic control. For instance, in the feature association task, participants selected the feature associate of a probe word (e.g., the item related in colour or size). In the low control manipulation (congruent trials), probe and target shared a semantic relationship (e.g., “*which is the same colour as cloud*,” target: *snow*), and the distractors were not semantically related to the probe (e.g., *egg*, *step*, *basket*). In the high control manipulation (incongruent trials), the probe and target did not share a semantic relationship, but one of the distractors was semantically related to the probe (e.g., probe = *salt*, colour associate = *dove*, distractor = *pepper*). Older adults displayed poorer accuracy and longer RTs on the semantically incongruent trials, compared to younger adults (Hoffman, 2018; Hoffman et al., 2018).

Hoffman and colleagues also found semantic control to be related to coherence during language production, with participants who performed poorly on the semantic control task also producing less coherent speech. In Study 2, we therefore examined potential relationships between semantic control and context effects (in particular, the Mismatch effect). We expected participants who exhibited poorer performance on measures of semantic control to exhibit a larger (negative) Mismatch effect (greater interference from competing semantic information).

#### Inhibitory control

Domain general inhibitory control has also been found to decline with age. For example, older adults have been found to experience greater interference costs than younger adults from incongruent stimuli in colour Stroop tasks, where participants are asked to produce a response based on the text colour of presented words while ignoring word meaning (e.g., when the word “red” is presented in green text; Spieler et al., 1996; West & Alain, 2000). Similar age effects have been found on other inhibition tasks too (e.g., Hoffman, 2018) although they might depend on the task used (e.g., de Bruin & Della Sala, 2018; Rey-Mermet et al., 2018).

Similar to semantic-specific control processes, domain-general inhibitory control processes may also influence language production by facilitating the suppression of competing words. This could be especially relevant for the Mismatch effect, where participants have to produce an unexpected word while controlling interference from an expected word. Indeed, poorer inhibition skills can predict the level of coherence in individuals’ speech (Arbuckle & Gold, 1993; Yin & Peng, 2016). In contrast, however, Higby et al. (2019) did not find a relationship between inhibition and picture or object-naming response times. In addition to semantic control, Study 2 therefore also assessed inhibition to examine whether poorer inhibition skills are associated with a larger Mismatch effect.

#### Short-term memory capacity

In line with resource theories, short-term memory capacity has been found to decline with age (Van der Linden et al., 1994; Waters & Caplan, 2003). For example, the mean number of items recalled in span tasks (including forward and backward digit, and letter and word span tasks) is lower in older adults (see Bopp & Verhaeghen, 2005, for a review). Short-term memory has also been associated with language processing (e.g., Feier & Gerstman, 1980; Kemper et al., 1989; Walsh & Baldwin, 1977). In Study 2, we therefore explored how age-related short-term memory declines (measured through the digit span task) may contribute to the Context effects (compared to no context). When producing words in context, speakers must hold the context within their working memory while planning and producing their verbal response. Producing words without context might not place the same demands on working memory resources. Thus, poorer working memory capacity might modulate how much participants can benefit from producing words in context, and therefore, the context effect in Study 1.

#### Lifestyle and social network

Finally, we included participants’ education, lifestyle, and social network in the analysis in Study 2. Education may serve as a protective factor that may reduce word-retrieval difficulties associated with ageing (Gordon & Kindred, 2011). Furthermore, lifestyle activities have been associated with reduced cognitive changes with age (Scarmeas et al., 2003). We furthermore assessed the frequency and nature of social interactions, as previous research suggests that language difficulties can be associated with reduced social interactions (Burke & Shafto, 2004; Farrell et al., 2014). Based on the described relationships between educational, lifestyle, and social factors and language, in Study 2, we were interested in exploring whether these factors also contribute to the context effects in Study 1.

#### Rationale Study 2

In Study 2, we assessed potential differences between younger and older adults on measures of language and cognitive functioning (including semantic knowledge, fluency, semantic control, inhibition, and verbal short-term memory capacity), as well as in terms of lifestyle and social interactions. We also studied how these variables related to language production in the different contexts assessed in Study 1. For the Match effect, we were particularly interested in semantic knowledge and fluency. For the mismatch context, although neither the younger nor the older adult group exhibited a mismatch effect in Study 1, we were interested in whether individual differences were linked to semantic control and/or inhibitory control. Finally, we assessed whether the magnitude of the context effect (neutral context versus naming in isolation) was related to individuals’ short-term memory capacity.

### Methods

This study was pre-registered on the Open Science Framework: https://osf.io/wf8cm. The data are available on https://osf.io/8qexr/, together with Study 1.

#### Participants

Ethical approval was obtained from the Department of Psychology at the University of York. All participants who completed Study 1 were invited again to complete Study 2, with 45 older and 38 younger adults taking part. Compared to the full set of participants in Study 1, the participant profile was comparable in terms of age (*M* younger adults = 24.61 years, *SD* = 4.79; *M* older adults = 69.09, *SD* = 4.12) and education (*M* younger adults = 16.57 years, *SD* = 2.99; *M* older adults = 15.60, *SD* = 3.36). The mean interval between completing Study 1 and Study 2 was 22.86 days (range = 6–45 days, *SD* = 7.65).

#### Materials/tasks

##### Semantic knowledge

Semantic knowledge was assessed through a synonym judgement task (adapted from the study by Wu & Hoffman, 2022). Participants viewed 67 word pairs and decided if the two words shared a similar meaning by pressing a keyboard button (S = related; D = different). Half of the word pairs were unrelated (e.g., “*formidable*,” “*obdurate*”), and the other half were related (“*recondite*,” “*abstruse*”). There was no time limit per trial, and the next trial started automatically when participants completed the previous trial. For each participant, we calculated an ISDT score (an index based on the signal detection theory) to correct for guessing and response style (Huibregtse et al., 2002). This score takes into consideration participants’ hit rate (proportion of correct “related” responses), as well as their false alarm rate (proportion of “related” responses to different pairs of words). This score ranges from 0 to 1 (a score closer to 1 indicates better performance, whereas a score closer to 0 indicates lower performance).

##### Verbal fluency

We measured both letter and semantic fluency. Participants completed three letter fluency trials (producing as many words as they could beginning with “F,” “A,” or “S”) and three semantic fluency trials (“animals,” “fruits,” and “items of clothing”). Each trial was 60 s long. For each participant, we computed a composite fluency score (the average of number of words produced across the letter and semantic trials). Fluency data from one older and one younger participant were excluded from the analyses because the recordings were of poor quality or empty.

##### Semantic control

Semantic control was measured through two semantic association tasks (used in Hoffman, 2018). In the global association task, participants selected the word associated with a probe, from a set of possible answers. Within the low demand condition (50% of trials), there was a strong semantic relationship between the probe and target (e.g., probe: *town*, target: *city*). In the high-demand condition, there was a weak semantic relationship between the probe and target (e.g., probe: *iron*, target: *ring*).

In the feature association task, participants selected the word matched with a probe word on a particular feature (e.g., colour or size, while ignoring distractor words). Half of the trials required participants to select the word most closely related in size, and the other half to select the word most closely related in colour to the probe. Half were congruent trials (target and probe shared a semantic relationship). For example, participants would see “which is the most similar in size to *door*?,” with “*window*” being the target and none of the distractors (*bottle*, *report*, *factory*) sharing a semantic relationship with the probe. In the incongruent feature trials, the target and probe were not semantically related, but one of the distractors was related to the probe. For example, participants would see “which is most similar in size to *ashtray?*” with the targets *diary* and distractor *cigarette*. There was no time limit (following the work of Hoffman, 2018), and the next trial started automatically when they completed the previous trial.

In both tasks, half of the trials included four answer options, and the other half, two; in the analyses, we collapsed across number of options. In the main analysis, we calculated the accuracy cost within each task for each participant. This was the z-scored accuracy difference between the low- and high-control conditions in the global association task (global cost) and the low- and high-control conditions in the feature association task (feature cost). Additional analyses were conducted using RTs, for which we removed RTs two standard deviations above or below participants’ conditional mean (following the work of Hoffman, 2018, and considering this task did not have a time limit), as well as incorrect responses.

##### Inhibitory control

Inhibitory control was measured through a verbal and non-verbal Stroop task. In the verbal version, participants provided a keyboard response based on the colour of a written word. In congruent trials (*n* = 28), word meaning and colour were the same (e.g., “red” presented in red), while they differed in incongruent trials (*n* = 28, e.g., the word “blue” presented in red). Neutral trials used a non-colour word (*n* = 28, e.g., “flower” presented in blue). The non-verbal version was a digit-based task, where participants decided which of two presented numbers was larger physically in terms of text size while ignoring numerical size. In the congruent trials (*n* = 28), the number which was physically bigger was also numerically bigger than the other number. In the incongruent trials (*n* = 28), the numerically smaller number was presented in a larger font. In neutral trials (*n* = 28), the same number was presented twice.

In each trial, participants saw a fixation cross for 500 ms, followed by the stimulus. The next trial was presented as soon as a response was given or, if no response was provided, after 3,000 ms.

Verbal and non-verbal Stroop interference costs were computed for each participant as the mean RT difference between the neutral and incongruent trials in each task. By computing the difference between neutral and incongruent trials, we specifically looked at interference, leaving out the influence of facilitation on congruent trials. Prior to calculating Stroop cost, we first removed incorrect responses and RTs 2.5 *SD*s above or below the mean per condition and per participant. Participants’ composite Stroop cost was calculated by computing the mean cost across the two tasks. The colour Stroop data from one older participant was not saved successfully and was therefore excluded from analysis.

##### Short-term memory span

Short-term memory was assessed using a digit span task. Participants viewed sequences of two to eight digits, with the sequence size increasing after every two consecutive trials (16 trials in total). Within each trial, participants viewed a fixation cross for 250 ms, followed by the individual presentation of each digit. Digits were presented in the centre of the screen for 800 ms before the screen automatically proceeded to the next digit. At the end of each trial, a text box appeared, and participants were asked to type all the digits they could remember from that trial. We calculated the proportion of correct sequences recalled by each participant. All participants saw all sequences, even if they made mistakes earlier on in the task (with shorter sequences).

##### Lifestyle and social network scores

Demographic details were derived from two questionnaires (administered in Study 1 and Study 2). Four participants did not provide the total number of years of formal education they had received. A lifestyle questionnaire (adapted from Scarmeas et al., 2003) asked participants to state for a range of activities (e.g., reading or travelling, 18 items) on a scale from 1 (*never*) to 3 (*often*) how often they did them during the year preceding the Covid-19 pandemic (the study was conducted during 2021, when many Covid-19 social-distancing restrictions were still in place). Participants’ total score on the questionnaire was computed as their “lifestyle score” (minimum score = 18, maximum score = 54). In addition, we assessed social network size by asking participants to estimate the number of people they had had regular contact with in the past 6 months (including face-to-face, by phone or mail, or on the internet). This was assessed across categories including close friends, family, neighbours, co-workers, school/child relations, people who provide a service, and others. We added together the total number of people in each category to compute each participant’s social network size (adapted from the study by Bruine de Bruin et al., 2020). We removed “social network size” from the analysis for participants who reported having been in regular contact with more than 1,000 people in the past 6 months (*n* = 3).

#### Procedure

The experiment was conducted using Gorilla.sc (Anwyl-Irvine et al., 2020). Participants read a study information sheet and completed a consent form to confirm that they met the study criteria and agreed to participate. Participants also completed a sound check to ensure their microphone was working (required for the fluency task). After this, participants completed the tasks in the following order: synonym judgement, semantic control tasks, Stroop (verbal), Stroop (digit), digit span, letter and semantic fluency, and lifestyle/social questionnaire. The study took approximately 45–60 min.

#### Data analysis

First, we examined whether the age groups differed in their performance on these tasks, using independent *t*-tests. Next, we conducted three regression analyses assessing if the measures described above explained the Match, Mismatch, and Context effects observed in Study 1. We z-scored the data for each continuous predictor variable (across age groups, given that part of our analysis aimed to examine individual differences in relation to language/cognitive abilities across age). Pearson’s correlation coefficients between the predictors (all below .5) as well as variance inflation factor (VIF) statistics (VIF values < 2.5 for all predictors) suggested there were no multi-collinearity issues.

We conducted hierarchical multiple linear regression analyses with the Match, Mismatch, and Context effects as outcomes. In each model, demographic variables were inputted first (age, lifestyle score, gender, social network size, years of formal education); this was followed by the cognitive and language variables (synonym judgement ISDT score, fluency composite score, semantic control global cost, semantic control feature cost, Stroop composite interference cost, and digit span accuracy), and finally, we also included interactions between the cognitive/language variables and age. Although all participants completed all tasks, there were some technical issues with some data files, and we therefore removed missing data pairwise in the analyses. We computed split-half reliability estimates for the cognitive variables entered into the regression models. These were generally moderate (Spearman-Brown scores ranging between .43 and .74).

### Results

#### Age group comparisons

In the following part of the article, we first present the analyses comparing the age groups. In terms of semantic knowledge and verbal fluency, older adults outperformed the younger adults on the synonym judgement task (*M* older adults = .66, *SD* = .13, *M* younger adults = .47, *SD* = .16; *t*(81) = 6.169, *p* < .001, *d* = 1.359; see Supplementary Figure 1) and the verbal fluency task (*M* older adults = 18.37, *SD* = 3.35, *M* younger adults = 16.95, *SD* = 2.86; *t*(79) = 2.037, *p* = .045, *d* = 0.454, see Supplementary Figure 2).

In terms of semantic control (see Supplementary Figure 3), our pre-registration focused on accuracy scores. For both tasks, we computed a semantic control score by taking the difference between strong and weak trials (global association) and congruent and incongruent trials (feature association). In the global association task, older adults’ semantic control cost (*M* = 4.35%, *SD* = 8.61) was, surprisingly, significantly *lower* than the younger adults’ cost (*M* = 11.40%, *SD* = 6.76; *t*(81) = −4.093, *p* < .001, *d* = −0.902). In the feature association task, the numerical pattern went in the same direction but was not significant (older adults *M* = 12.78%, *SD* = 19.67; younger adults *M* = 17.11%, *SD* = 23.35; *t*(81) = −0.917, *p* = .362, *d* = −0.202). The RT data showed no significant cost difference in the global association task (older adults *M* = 836.24 ms, *SD* = 478.02; younger adults *M* = 699.74 ms, *SD* = 487.26; *t*(81) = 1.285, *p* = .203, *d* = 0.283). In the feature association task, the RT cost was significantly higher in the older (*M* = 1277.61 ms, *SD* = 815.00) than in the younger age group (*M* = 703.84 ms, *SD* = 735.63; *t*(80) = 3.325, *p* = .001, *d* = 0.736). Similar RT results were obtained when comparing the z-scored RTs, considering older adults responded more slowly overall. Given the potential speed-accuracy trade off (older adults showing larger RT costs with smaller accuracy costs), we also computed inverse efficiency scores (RT/percentage correct). These did not differ between age groups for the global task (*t*(81) = −.267, *p* = .790, *d* = −0.059) or for the feature task (*t*(80) = −.497, *p* = .620, *d* = −0.110).

The Stroop analysis, as pre-registered, focused on the RTs only. This analysis excluded incorrect responses. Accuracy in the non-verbal Stroop task was 82.07% (*SD* = 4.66) for older adults and 80.03% (*SD* = 9.19) for younger adults. In the verbal Stroop task, accuracy was 74.73% (*SD* = 13.97) for older adults and 81.21% (*SD* = 4.79) for younger adults. The Stroop RT interference cost was significantly larger in older adults (*M* Stroop = 82.41 ms, *SD* = 80.29) than in younger adults (*M* = 39.54 ms, *SD* = 85.18; *t*(80) = 2.344, *p* = .022, *d* = 0.519; see Supplementary Figure 4). This significant difference remained when analysing the z-scored RTs (considering overall slower responses in older adults).

Short-term memory capacity (measured through the digit span task) did not differ between older adults (*M* = 65.56%, *SD* = 14.06) and younger adults (*M* = 65.41%, *SD* = 17.30; *t*(81) = 0.041, *p* = .967, *d* = 0.009; see Supplementary Figure 5).

Finally, social network size was slightly larger in younger participants (*M* = 33.03, *SD* = 23.09) than that in older participants (*M* = 27.73, *SD* = 17.25), but this difference was not significant (*t*(78) = −1.174, *p* = .244, *d* = −0.264). Older adults scored significantly higher on the lifestyle questionnaire (*M* = 37.64, *SD* = 3.93) than younger participants (*M* = 34.66, *SD* = 3.84; *t*(81) = 3.483, *p* < .001, *d* = 0.767).

#### Hierarchical regression

Hierarchical linear regressions were computed next to measure the contribution of each predictor on each context effect from Study 1 (Match, Mismatch, and Context effect). Supplementary Figures 6–8 show the relationships between the synonym judgement, verbal fluency, the two semantic control (global and feature association), inhibition, and digit span tasks with the Match, Mismatch, and Context effects.

##### Match effect

None of the included cognitive variables were significant individual predictors of the Match effect (see Table 7). The contribution of demographic variables together (Model 1) was close to significance, with social network reaching significance. This suggests that people with a larger social network showed a smaller Match effect. Models 2 and 3, which included the cognitive and language variables and age interactions with those cognitive and language variables, did not contribute significantly beyond the model only including demographic variables.

**Table 7.** Hierarchical regression table showing the contributions of all predictors to the Match effect.

Model 1				Model 2				Model 3			
	*B*	*SE*	*p*		*B*	*SE*	*p*		*B*	*SE*	*p*
Intercept	−0.54	0.04	<.001	Intercept	−0.55	0.04	<.001	Intercept	−0.52	0.06	<.001
Age	−0.02	0.03	.632	Age	<−0.001	0.04	.999	Age	0.02	0.05	.666
Education	−0.04	0.03	.164	Education	−0.04	0.03	.219	Education	−0.03	0.04	.332
Lifestyle	0.06	0.03	.059	Lifestyle	0.07	0.04	.057	Lifestyle	0.06	0.04	.111
Social net	−0.07	0.03	.035	Social net	−0.07	0.03	.034	Social net	−0.07	0.04	.068
Gender	−0.10	0.06	.106	Gender	−0.08	0.07	.226	Gender	−0.09	0.07	.175
				Synonym judgement	−0.001	0.04	.981	Synonym judgement	−0.02	0.05	.716
				Fluency	−0.04	0.04	.292	Fluency	−0.02	0.04	.556
				Global cost	−0.01	0.04	.751	Global cost	−0.03	0.04	.534
				Feature cost	0.01	0.04	.860	Feature cost	−0.01	0.04	.893
				Inhibition	−0.01	0.04	.864	Inhibition	−0.02	0.04	.546
				Digit span	0.01	0.03	.677	Digit span	0.003	0.03	.918
								Age × Synonym judgement	−0.07	0.05	.148
								Age × Fluency	−0.03	0.04	.459
								Age × Global cost	0.07	0.05	.143
								Age × Feature cost	−0.01	0.04	.756
								Age × Inhibition	−0.02	0.04	.496
								Age × Digit span	−0.02	0.03	.627
Model 1: (*F*(5,70) = 2.252, *p* = .059) Total variance explained: 13.9%				Model 2: (*FChange*(6, 64) = .328, *p* = .920) Variance explained relative to Model 1: 2.6%				Model 3: (*FChange*(6, 58) = .850, *p* = .537) Variance explained relative to Model 2: 6.8%			

Global cost = accuracy difference between strong and weak trials within the global association task, Feature cost = accuracy difference between the congruent and incongruent trials within the feature association task.

##### Mismatch effect

The mismatch effect was not explained significantly by any of the individual predictors. None of the three models reached significance either (see Table 8)

**Table 8.** Hierarchical regression table showing the contributions of the predictor variables to the Mismatch effect.

Model 1				Model 2				Model 3			
	*B*	*SE*	*p*		*B*	*SE*	*p*		*B*	*SE*	*p*
Intercept	0.03	0.03	.281	Intercept	0.03	0.03	.393	Intercept	0.06	0.04	.102
Age	0.02	0.02	.495	Age	0.03	0.03	.319	Age	0.03	0.03	.413
Education	−0.02	0.02	.432	Education	−0.02	0.02	.349	Education	−0.03	0.02	.211
Lifestyle	0.01	0.02	.626	Lifestyle	0.01	0.02	.798	Lifestyle	0.003	0.03	.904
Social net	−0.001	0.02	.964	Social net	−0.002	0.02	.919	Social net	0.01	0.02	.599
Gender	−0.08	0.04	.059	Gender	−0.07	0.04	.122	Gender	−0.08	0.05	.078
				Synonym judgement	0.02	0.03	.418	Synonym judgement	0.01	0.03	.662
				Fluency	−0.01	0.02	.657	Fluency	0.002	0.03	.940
				Global cost	−0.05	0.03	.103	Global cost	−0.04	0.03	.136
				Feature cost	−0.003	0.02	.915	Feature cost	−0.01	0.03	.603
				Inhibition	−0.01	0.02	.726	Inhibition	−0.01	0.03	.653
				Digit span	−0.02	0.02	.485	Digit span	−0.02	0.02	.403
								Age × Synonym judgement	−0.02	0.03	.478
								Age × Fluency	−0.03	0.02	.238
								Age × Global cost	−0.04	0.03	.299
								Age × Feature cost	−0.03	0.03	.276
								Age × Inhibition	0.01	0.02	.765
								Age × Digit span	0.001	0.02	.947
Model 1 Stats: (*F*(5,70) = 1.077, *p* = .381 Total variance explained: 7.1%				Model 2 Stats: (*FChange*(6,64) = .866, *p* = .525 Variance explained relative to Model 1: 7.0%				Model 3 Stats: (*FChange*(6,58) = .790, *p* = .582) Variance explained relative to Model 2: 6.5%			

Global cost = accuracy difference between strong and weak trials within the global association task, Feature cost = accuracy difference between the congruent and incongruent trials within the feature association task.

##### Context effect

Finally, none of the included variables were significant predictors of the Context effect (see Table 9). None of the three overall models reached significance either.^1^

**Table 9.** Hierarchical regression table showing the contributions of the predictors to the Context effect.

Model 1				Model 2				Model 3			
	*B*	*SE*	*p*		*B*	*SE*	*p*		*B*	*SE*	*p*
Intercept	−0.22	0.10	.024	Intercept	−0.22	0.10	.029	Intercept	−0.13	0.13	.327
Age	0.12	0.08	.120	Age	0.05	0.10	.623	Age	0.01	0.10	.953
Education	0.06	0.07	.422	Education	0.05	0.07	.519	Education	0.02	0.08	.821
Lifestyle	−0.10	0.08	.180	Lifestyle	−0.07	0.08	.372	Lifestyle	−0.05	0.09	.599
Social net	0.10	0.07	.183	Social net	0.10	0.07	.194	Social net	0.12	0.08	.132
Gender	0.12	0.14	.380	Gender	0.12	0.15	.401	Gender	0.12	0.15	.439
				Synonym judgement	0.05	0.10	.602	Synonym judgement	0.03	0.11	.807
				Fluency	−0.05	0.08	.548	Fluency	−0.03	0.08	.729
				Global cost	0.04	0.10	.697	Global cost	0.06	0.10	.548
				Feature cost	0.02	0.08	.785	Feature cost	0.02	0.09	.807
				Inhibition	0.09	0.08	.248	Inhibition	0.10	0.08	.224
				Digit span	−0.04	0.07	.567	Digit span	−0.07	0.08	.372
								Age × Synonym judgement	−0.03	0.11	.769
								Age × Fluency	−0.01	0.08	.909
								Age × Global cost	−0.17	0.11	.136
								Age × Feature cost	0.003	0.09	.974
								Age × Inhibition	0.01	0.08	.949
								Age × Digit span	−0.05	0.07	.531
Model 1 Stats: (*F*(5, 70) = .967, *p* = .444) Total variance explained: 6.5%			Model 2 Stats: (*FChange*(6, 64) = .553, *p* = .766) Variance explained relative to Model 1: 4.6%					Model 3 Stats: *(FChange*(6, 58) = .670, *p* = .674) Variance explained relative to Model 2: 5.8%			

Global cost = accuracy difference between strong and weak trials within the global association task, Feature cost = accuracy difference between the congruent and incongruent trials within the feature association task.

### Discussion

In Study 2, we assessed how the older and younger adults tested in Study 1 differed on various cognitive abilities including semantic knowledge and control, inhibition, and short-term working memory capacity. Semantic knowledge and fluency scores were larger for older adults than for younger adults. However, older adults performed more poorly in terms of inhibition costs and (some aspects of) semantic control, although they did outperform younger adults in terms of semantic control accuracy. The language and cognitive variables assessed did not relate to language-production Match, Mismatch, or Context effects tested in Study 1. This will be discussed further in the general Discussion.

#### Semantic knowledge

Corroborating previous research (e.g., Carrol, 2023; Hoffman, 2018; Hoffman et al., 2018; Kavé & Halamish, 2015; Kavé & Yafé, 2014; Verhaeghen, 2003), older adults performed significantly better on the synonym judgement task than the younger adults, suggesting that semantic knowledge was higher in the older than younger adults. Furthermore, composite verbal fluency was also higher in the older adult group. In line with previous research (e.g., Gordon et al., 2018), exploratory analyses presented in the Supplementary Materials showed this benefit for older adults was driven by the letter fluency trials rather than the semantic fluency trials. This aligns with a frequently, although seemingly paradoxically, observed pattern in the literature reflecting older adults are more likely to experience difficulties on the semantic than letter fluency task (cf. Gordon et al., 2018). Although letter fluency is often associated with executive control and would therefore be expected to be influenced more strongly by age, previous research has suggested letter fluency relies more heavily on vocabulary knowledge. The finding that our older adults outperformed the younger adults on this fluency task specifically supports, in line with the synonym judgement task and the literature, the interpretation that older adults continue to benefit from their (larger) vocabulary knowledge. In contrast, semantic fluency has been found to be more heavily influenced by lexical retrieval speed (Gordon et al., 2018). Indeed, our supplementary analyses showed no significant age-group difference here. If anything, older adults performed a little worse than younger adults on this task.

#### Semantic and domain general control

To measure semantic control, we used global association and feature association tasks (Hoffman, 2018). Both showed costs (poorer accuracy and longer RTs) in the conditions associated with higher control demands. In terms of accuracy, both measures showed higher costs in younger adults than in older adults, although this difference was not statistically significant in the feature association task. These accuracy effects were contrary to our predictions regarding older adults experiencing difficulties with semantic control (and the findings observed in the study by Hoffman, 2018). Some previous research has reported age-related increases in motivation and engagement with lab-based tasks (Frank et al., 2015; Jackson & Balota, 2012). This could explain why older adults were less hindered by the more challenging conditions in the semantic control tasks, especially given that there was no time constraint within these tasks. On the other hand, older adults did exhibit a greater RT cost, although only on the feature association measure. The feature association task was the task showing the largest accuracy and RT costs across age groups, suggesting older adults showed larger RT costs only on the more-demanding control task. The combination of accuracy and RT findings suggests the older adults needed more time during the semantic-control task to suppress irrelevant features but were able to achieve higher accuracy by doing this. Older adults also showed a larger interference Stroop cost. This was especially the case in the verbal Stroop task, where older adults also showed lower accuracy than younger adults (suggesting this larger RT cost was not due to a speed-accuracy trade off). These findings suggest older adults showed poorer semantic (on some measures) and inhibitory control in terms of response times, lending support to the inhibition-deficit hypothesis (cf. also Hoffman, 2018).

#### Working memory

Finally, working memory capacity did not differ between the two age groups. Forward digit span tasks measuring storage capacity might not be sufficiently sensitive to age-related declines in working memory, relative to tasks that focus on both storage and information manipulation such as the backward digit span task (e.g., Babcock & Salthouse, 1990; Bopp & Verhaeghen, 2005). Furthermore, to adjust the task to an online environment, our participants were able to continue onto longer sequences even if they made errors in shorter trials, which may have influenced performance on this task relative to traditional task versions not allowing this.

#### Lifestyle and social network

Older and younger adults showed some differences in terms of their lifestyle and social network. Older adults reported higher engagement in the lifestyle activities we assessed than the younger adults, although this may partly be related to the type of activities assessed (e.g., gardening). Social network size did not differ significantly between the age groups.

## General discussion and conclusion

Together, Studies 1 and 2 show that both younger and older adults’ language production is influenced by the sentence context preceding a target word. Semantically matching contexts facilitated both younger and older adults’ lexical retrieval. These context effects can be explained through more automatic mechanisms such as semantically related words being primed by previously presented words, therefore facilitating lexical retrieval during production. In this specific task context, where participants had to alternate between comprehending speech and producing a response, this priming operated cross-modally. Linked to priming, speakers can use the speech presented to them to predict suitable upcoming responses to their conversation partner. Our study shows that both younger and older adults continue to use sentence context to facilitate their own responses, with no significant difference between the groups. This suggests older adults may continue to use their semantic knowledge to facilitate lexical retrieval during production.

These findings support theories arguing that semantic knowledge is more developed in later adulthood, and that this can benefit language-production processes (Burke et al., 1991). The transmission deficit hypothesis argues that older adults’ slower lexical retrieval is related to slower connections between the lexical and phonological levels, with the semantic level staying intact. Our findings, in particular regarding the Match effect, support this hypothesis and suggest the preservation or proliferation of representations within the semantic system can facilitate connections between the semantic and lexical level. Our study did also reveal an age-group difference in terms of overall RTs, suggesting (in line with the Transmission Deficit Hypothesis and the general literature) that overall production was slower in older adults. This could be related to older adults showing slower lexical retrieval; however, without specifically examining lexical-phonological retrieval difficulties in the current study, it might also reflect general processing speed.

Although individual differences in participants’ semantic knowledge and fluency were not directly related to the size of the Match effect (Study 2), it is possible that a certain degree of semantic knowledge is a prerequisite for speakers to benefit from matching contexts. With our older adults on average outperforming the younger adults on semantic knowledge and fluency tests, this prerequisite seemed to have been met by the older adults as a group and almost all older adults at the individual level. Indeed, only four of the older adults’ semantic knowledge scores fell below the mean score for the younger adults. However, in the absence of a direct relationship between semantic knowledge and the Match effect, the exact contribution of semantic knowledge in older adults requires further research.

The finding that older adults showed the same Match (and no Mismatch) effect as younger adults while showing more semantic knowledge argues against previous research suggesting increased (semantic) knowledge in older adults could potentially create more interference (e.g., Ramscar et al., 2014, cf. Hoffman et al., 2018, showing adults with greater semantic knowledge were less coherent in connected speech). If larger semantic knowledge is associated with greater interference, our older adults should have shown a smaller Match (and potentially larger Mismatch) effect than younger adults at the group level.

Finally, it is worth further exploring the role of lifestyle variables in relation to semantic connections in sentence contexts. Social network size predicted the Match effect during language production. People with a smaller social network showed greater facilitation from matched contexts. This relationship was small and not significant in all models and does therefore require further research. It is, however, in line with previous research suggesting a relationship between social interactions and language difficulties (Farrell et al., 2014).

Contrary to our expectations, neither age group showed an interference cost during mismatching contexts (that were designed to prime a word other than the target). Furthermore, neither semantic nor inhibition costs (measured in Study 2) were significant predictors of the Mismatch effect in the main analysis. Although some previous studies have shown a relationship between semantic and inhibitory control and certain aspects of language production (e.g., Hoffman et al., 2018), our findings align with previous research showing no direct relationship between inhibition and picture naming times in younger and older adults (Higby et al., 2019). This could suggest that measures related to the types of words older adults use (e.g., speech coherence, Hoffman et al., 2018) could be more closely related to one’s ability to suppress interfering information than speed of lexical retrieval (as examined in the study by Higby et al., 2019). We did also expect such a relationship (with semantic and inhibitory control) to arise in our mismatch contexts, which specifically required the production of an unexpected rather than expected target word. With this Mismatch effect not arising at the group level in either age group, however, it is very likely that a stronger mismatching sentence context is necessary for any role of semantic or inhibitory control abilities to emerge.

Study 2 did show that older adults’ responses were slower during high-control trials in both semantic and inhibitory control tasks, corroborating previous findings that these cognitive abilities can decline in old age (e.g., Hoffman, 2018; Spieler et al., 1996). This suggests that the absence of a Mismatch effect in the group of older adults was not the consequence of recruiting a sample of older adults with particularly high or fully preserved semantic or inhibitory control. Rather, it suggests that interference in the mismatched sentences might have been too weak to lead to a noticeable impact on language production. Future research will need to study the potential relationship between age-related changes in inhibitory or semantic control and stronger context violations during language production. Furthermore, such research might want to include older adults with more difficulties in terms of their semantic and inhibitory control. While age-group differences were observed in the current study, they were not present on all semantic control tasks and measures, and older adults might have slowed down their responses to achieve a higher accuracy level. As is common in these types of studies, the older adults (like the younger adults) had a relatively high level of education and a relatively active lifestyle. Research including a wider range of older adults from various backgrounds, including lower socio-economic status, would be more representative of the general population and might be more likely to capture age-related changes in terms of interference effects during cognitive and language-production tasks. Including a larger sample size would also be beneficial, as the current sample size might have limited the power to detect effects related to individual differences in Study 2. Furthermore, the older adults in our study were quite young (mean age < 70 years). Future research might also want to include older adults with a higher age, including more participants over 75 years old. It is important to underline, however, that despite not observing the age-related changes in the context effects we investigated, in Study 1, we did observe the expected age-related slowing in older adults’ overall naming RTs. This is in line with data that show that RTs are sensitive to age-related changes in language production from the age of 50 years (Verhaegen & Poncelet, 2013).

Finally, previous research comparing age-group differences during language production with and without context has suggested any age effects might be less likely to be observed during connected speech in context (Kavé & Goral, 2017). However, such comparison across studies is made more difficult by the range of measures used (e.g., hesitations, naming times, circumlocutions). Furthermore, in connected speech, older adults can use compensatory mechanisms to mask lexical-retrieval difficulties. The current study therefore compared language production in older and younger adults in the form of picture naming with and without sentence context. This allowed us to compare the same measure (naming times) and removed any compensatory strategies that could be used during free speech. If older adults’ lexical-retrieval difficulties are reduced during context, context effects (faster picture naming in a sentence context) should have been larger for older than younger adults. That was not the case; if anything, some analyses suggested the context effect was smaller in older adults. In combination with older adults not benefitting more from matched sentence contexts, this suggests that previously observed lexical-retrieval difficulties in older adults in picture-naming tasks are not purely due to somewhat artificial tasks requiring production of words without any context.

While the current study included producing words in response to a preceding question (as is common behaviour when people respond to other speakers), we did not examine a speaker’s lexical retrieval within their own connected speech. Therefore, future research is needed to better understand the various mechanisms older adults might use when retrieving words in connected speech during different types of contexts. Context effects in the current study were not related to any of the language or cognitive abilities tested, including digit-span performance. This suggests that keeping the question in mind in a context as compared to naming pictures in isolation was not modulated by working memory capacity as assessed through this task. Sentences in our study were relatively short and simple. It is possible that more complex sentences, or producing words in connected speech and interactions with others, do tax short-term memory capacity more strongly (e.g., Kemper, 1986).

In conclusion, our findings suggest that the contexts within which words are produced have significant consequences for lexical-retrieval efficiency. Our study provides an important first step in comparing word production with and without context, while using the same measure (naming response times) to bridge the gap between existing literatures. Within the context of cognitive ageing, our findings highlight the important role of preserved semantic networks within older adults who can continue to benefit from context when retrieving words to respond to questions asked by their conversation partner.

## Supplemental Material

sj-docx-1-qjp-10.1177_17470218241309602 – Supplemental material for Naming speed during language production in younger and older adults: Examining the effects of sentence context Supplemental material, sj-docx-1-qjp-10.1177_17470218241309602 for Naming speed during language production in younger and older adults: Examining the effects of sentence context by Naveen Hanif, Elizabeth Jefferies and Angela de Bruin in Quarterly Journal of Experimental Psychology

## Notes

1. Considering that (negative) age effects on the semantic control task were observed in terms of RTs but not accuracy, we re-ran the regression analyses with the RT global and feature association costs. These analyses did not find significant effects of the included cognitive predictors on Matched, Mismatched, or Context effects either, with the exception of Age × Global RT cost being a predictor of the Mismatch effect. For younger adults, a larger semantic-control cost was associated with a slightly smaller Mismatch effect, while for older adults, a larger semantic-control cost was associated with a slightly larger Mismatch effect. However, this control cost was not a significant predictor for either age group in separate regression analyses per group. Finally, we checked whether any of the cognitive and language scores related significantly to overall naming times (as opposed to the context effects specifically). Analyses using RTs in the no-context task showed no relationship with any of the predictors either.
