# Supplementary material for: Naming speed during language production in younger and older adults: Examining the effects of sentence context
Source: Q J Exp Psychol (Hove). 2025 Jan 8;78(11):2355–78. doi: 10.1177/17470218241309602 (PMC12531402; doi:10.1177/17470218241309602)
Supplement: sj-docx-1-qjp-10.1177_17470218241309602 – Supplemental material for Naming speed during language production in younger and older adults: Examining the effects of sentence context [file sj-docx-1-qjp-10.1177_17470218241309602.docx]

Supplementary Materials for:

**Naming speed during language production in younger and older adults: Examining the**

**effects of sentence context**

Naveen Hanif^1^, Elizabeth Jefferies^1^, & Angela de Bruin^1^

^1^University of York

Corresponding author:

Angela de Bruin

Department of Psychology

University of York

York

YO10 5DD

[angela.debruin@york.ac.uk](mailto:Angela.debruin@york.ac.uk)

1. **Stimuli**

Stimuli were divided into two sets. Sentences that formed a “matched” context for one target in Set 1 were a “mismatched” context for another target in Set 2. The neutral sentences were the same in the two sets. Participants either named the Set 1 targets or the Set 2 targets.

Although Sets 1 and 2 were not compared, we also ensured the target words in the two sets were comparable in terms of log frequency (NWatch, Davis, 2005; Set 1: *M* = 1.577, *SD* = 0.547; Set 2: *M* = 1.458, *SD* = 0.601; *t*(78) = 0.931, *p* = .355); number of syllables (Set 1: *M* = 1.500, *SD* = 0.679; Set 2: *M* = 1.525, *SD* = 0.679; *t*(78) = -0.165, *p* = .870); and number of phonemes (Set 1: *M* = 4.150, *SD* = 1.511; Set 2: *M* = 4.175, *SD* = 1.412; *t*(78) = -0.076, *p* = .939).

The sentences were also matched in terms of number of words between the matched/mismatched sentences (*M* = 6.412, *SD* = 1.651) and neutral sentences (*M* = 6.525, *SD* = 1.867; *t*(118) = -0.337, *p* = .737). Within each sentence we identified the key words (all sentence subjects, nouns, verbs, and adjectives). Mean log frequency and number of syllables of the key words (averaged across the key words per sentence) were also comparable between the matched/mismatched sentences (*M* frequency = 1.772, *SD* = 0.622; *M* syllables = 1.578, *SD* = 0.602) and neutral sentences (*M* frequency = 1.909, *SD* = 0.548; *M* syllables = 1.772, *SD* = 0.582; frequency: *t*(118) = -1.181, *p* = .240; syllable length: *t*(118) = -1.686, *p* = .094)

| **Set 1** | | | | |
| --- | --- | --- | --- | --- |
|  | **Targets** | **MATCHED** | **MISMATCHED** | **NEUTRAL** |
| **1** | Present | What did she wrap before Christmas? | What did the football player break? | What did grandfather perceive? |
| **2** | Potato | What did the dinner lady mash? | What did the monkey eat? | What did the cat examine? |
| **3** | Knife | What was the defendant accused of stabbing the victim with? | What did the dog lick? | What was the young man holding? |
| **4** | Ring | What do married women wear? | What did the queen wear? | What did the girl lift off the table? |
| **5** | Bible | What did the priest read? | What did the father read to his daughter before bed? | What did the man pick up? |
| **6** | Lamp | What did she turn off before bed? | What did the kitchen assistant switch on before preparing dinner? | What did the man use during the day? |
| **7** | Rose | What did he give to his date? | What did the thief steal? | What did the girl request? |
| **8** | Bin | What did they put the rubbish into? | What did the little girl put the sand into? | What did the children cleanse? |
| **9** | Ice | What did he add to the cold drink? | What did the sweet pastry chef melt? | What did the assistant pour? |
| **10** | Ship | What did the captain navigate back to the harbour? | What did the pilot crash? | What did the character destroy? |
| **11** | Duck | What did the toddler feed the bread to? | Who was the new mum feeding? | What distracted the little girl away from her toys? |
| **12** | Water | What did he spill? | What did the baker pour into the jar? | What did the student chill for a few hours? |
| **13** | Bottle | What did they store the water in? | What did the commuter drink coffee from? | What did the guy handle? |
| **14** | Fireworks | What was the dog frightened of? | What did she light on the dinner table? | What did they put in the box? |
| **15** | Bread | What did the baker make with yeast? | What did she eat on her birthday? | What did the pupil make in her spare time? |
| **16** | Mountain | What did the alpinist climb? | What did the construction worker climb? | What did they reach? |
| **17** | Bandage | What did he wrap around the wound? | What did the midwife wrap the newborn in? | What did the consumer snatch from the man? |
| **18** | Horse | What did the jockey ride? | What did she ride to work? | What object did he win in the competition? |
| **19** | Calendar | Where did mum write the scheduled family events? | What did the artist illustrate? | What did the lady create? |
| **20** | Hair | What did the barber chop? | What did the butcher chop? | What did she inspect vigilantly? |
| **21** | Carrot | What did the rabbit eat? | What did the mouse nibble on? | What did the associates purchase for their endeavour? |
| **22** | Purse | What did she carry her money in? | What did she keep the cookies in? | What did the onlooker have? |
| **23** | Painting | What did the artist admire? | What did the seamstress create? | What did the attendee comment on at the event? |
| **24** | Bag | Where did the athlete keep his kit? | What did she put the sleeping baby into? | What did the guest fix at the weekend? |
| **25** | Bed | Where did he sleep? | What did the automobile salesman sell? | What did the visitor purchase at the establishment? |
| **26** | Egg | What did the chef crack? | What did the baby shake? | What did the father ask his son to bring? |
| **27** | Bank | Which institute did the thief rob? | Where did the mum drop off her daughter? | Which organisation did the gentleman contact to discuss the matter? |
| **28** | Tenant | Who did the landlord evict? | Who drew the sketch? | Who did the team get in touch with? |
| **29** | Beer | What did the student purchase at the bar? | What did grandma sweeten? | What did grandpa discard? |
| **30** | Ball | What did the school children play with? | What did the pirate discover? | What did the children share? |
| **31** | Tree | What did the squirrel climb? | What was the guard dog sitting by? | What did the committee remove? |
| **32** | Rope | What did the sailor knot? | What was the little boy learning to tie? | What did they forget to put in the bag? |
| **33** | Wood | What did the carpenter cut? | What did the beautician file? | What did the girl damage? |
| **34** | Glue | What did he use to stick the pieces of paper together? | What was the kid chewing? | What did the teenager retrieve from the box? |
| **35** | Finger | What did he put the ring on? | What did the gardener cut? | What did the new occupant find? |
| **36** | Chimney | What was the smoke coming out of? | What did the maid sweep? | What did the guardian assess? |
| **37** | Prisoner | Who did the guard escort back to the cell? | Who did the doctor treat? | Who did the spectator meet? |
| **38** | Gardener | Who was trimming the hedge? | Who did the resident alert? | Who did the candidate consult? |
| **39** | Tin | What were the baked beans stored in? | What did she ladle the soup into? | What did the parent hand over to the supervisor? |
| **40** | Hand | What did the candidate shake at the start of the interview? | What did she put the coat on? | What did she break? |

| **Set 2** | | | | |
| --- | --- | --- | --- | --- |
|  | **Targets** | **MATCHED** | **MISMATCHED** | **NEUTRAL** |
| **1** | Leg | What did the football player break? | What did she wrap before Christmas? | What did grandfather perceive? |
| **2** | Banana | What did the monkey eat? | What did the dinner lady mash? | What did the cat examine? |
| **3** | Bone | What did the dog lick? | What was the defendant accused of stabbing the victim with? | What was the young man holding? |
| **4** | Crown | What did the queen wear? | What do married women wear? | What did the girl lift off the table? |
| **5** | Fairy tale | What did the father read to his daughter before bed? | What did the priest read? | What did the man pick up? |
| **6** | Oven | What did the kitchen assistant switch on before preparing dinner? | What did she turn off before bed? | What did the man use during the day? |
| **7** | Money | What did the thief steal? | What did he give to his date? | What did the girl request? |
| **8** | Bucket | What did the little girl put the sand into? | What did they put the rubbish into? | What did the children cleanse? |
| **9** | Butter | What did the sweet pastry chef melt? | What did he add to the cold drink? | What did the assistant pour? |
| **10** | Aeroplane | What did the pilot crash? | What did the captain navigate back to the harbour? | What did the character destroy? |
| **11** | Baby | Who was the new mum feeding? | What did the toddler feed the bread to? | What distracted the little girl away from her toys? |
| **12** | Jam | What did the baker pour into the jar? | What did he spill? | What did the student chill for a few hours? |
| **13** | Flask | What did the commuter drink coffee from? | What did they store the water in? | What did the guy handle? |
| **14** | Candle | What did she light on the dinner table? | What was the dog frightened of? | What did they put in the box? |
| **15** | Cake | What did she eat on her birthday? | What did the baker make with yeast? | What did the pupil make in her spare time? |
| **16** | Ladder | What did the construction worker climb? | What did the alpinist climb? | What did they reach? |
| **17** | Blanket | What did the midwife wrap the newborn in? | What did he wrap around the wound? | What did the consumer snatch from the man? |
| **18** | Bike | What did she ride to work? | What did the jockey ride? | What object did he win in the competition? |
| **19** | Book | What did the artist illustrate? | Where did mum write the scheduled family events? | What did the lady create? |
| **20** | Meat | What did the butcher chop? | What did the barber chop? | What did she inspect vigilantly? |
| **21** | Cheese | What did the mouse nibble on? | What did the rabbit eat? | What did the associates purchase for their endeavour? |
| **22** | Jar | What did she keep the cookies in? | What did she carry her money in? | What did the onlooker have? |
| **23** | Dress | What did the seamstress create? | What did the artist admire? | What did the attendee comment on at the event? |
| **24** | Cot | What did she put the sleeping baby into? | Where did the athlete keep his kit? | What did the guest fix at the weekend? |
| **25** | Car | What did the automobile salesman sell? | Where did he sleep? | What did the visitor purchase at the establishment? |
| **26** | Rattle | What did the baby shake? | What did the chef crack? | What did the father ask his son to bring? |
| **27** | School | Where did the mum drop off her daughter? | Which institute did the thief rob? | Which organisation did the gentleman contact to discuss the matter? |
| **28** | Artist | Who drew the sketch? | Who did the landlord evict? | Who did the team get in touch with? |
| **29** | Tea | What did grandma sweeten? | What did the student purchase at the bar? | What did grandpa discard? |
| **30** | Treasure | What did the pirate discover? | What did the school children play with? | What did the children share? |
| **31** | Door | What was the guard dog sitting by? | What did the squirrel climb? | What did the committee remove? |
| **32** | Laces | What was the little boy learning to tie? | What did the sailor knot? | What did they forget to put in the bag? |
| **33** | Nails | What did the beautician file? | What did the carpenter cut? | What did the girl damage? |
| **34** | Gum | What was the kid chewing? | What did he use to stick the pieces of paper together? | What did the teenager retrieve from the box? |
| **35** | Grass | What did the gardener cut? | What did he put the ring on? | What did the new occupant find? |
| **36** | Floor | What did the maid sweep? | What was the smoke coming out of? | What did the guardian assess? |
| **37** | Patient | Who did the doctor treat? | Who did the guard escort back to the cell? | Who did the spectator meet? |
| **38** | Policeman | Who did the resident alert? | Who was trimming the hedge? | Who did the candidate consult? |
| **39** | Bowl | What did she ladle the soup into? | What were the baked beans stored in? | What did the parent hand over to the supervisor? |
| **40** | Hook | What did she put the coat on? | What did the candidate shake at the start of the interview? | What did she break? |

**2. Supplementary graphs Study 2**

*p< 0.05 (two-tailed); **p< 0.01 level (two-tailed); ***p<.001 (two-tailed).


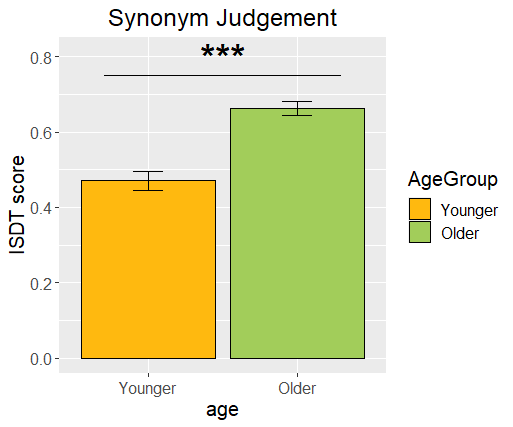


*Supplementary Figure 1.* Bar chart displaying the synonym judgement mean ISDT (considering hits and false alarms) score per age group in the synonym judgement task.


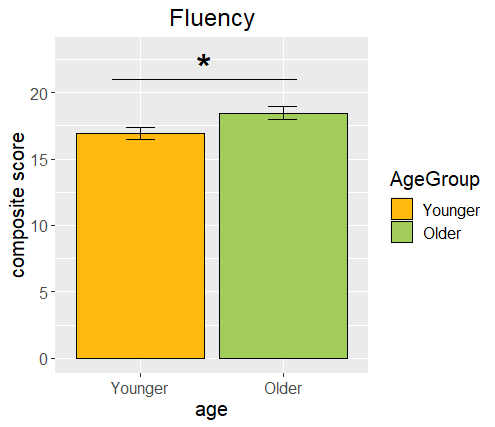


*Supplementary Figure 2.* Bar chart displaying the mean fluency composite score per age group in the verbal fluency task.

As pre-registered, the verbal fluency score used in the regression analysis was computed across the two tasks. However, further analyses showed the age-group difference was only significant for the letter fluency task (*M* older adults = 18, *SD* = 4; *M* younger adults = 14, *SD* = 3; t(79) = 4.538, p < .001). There was no significant difference on the semantic fluency task (*M* older adults = 19, *SD* = 4; *M* younger adults = 20, *SD* = 4; t(79) = -0.924, p = .358).

**
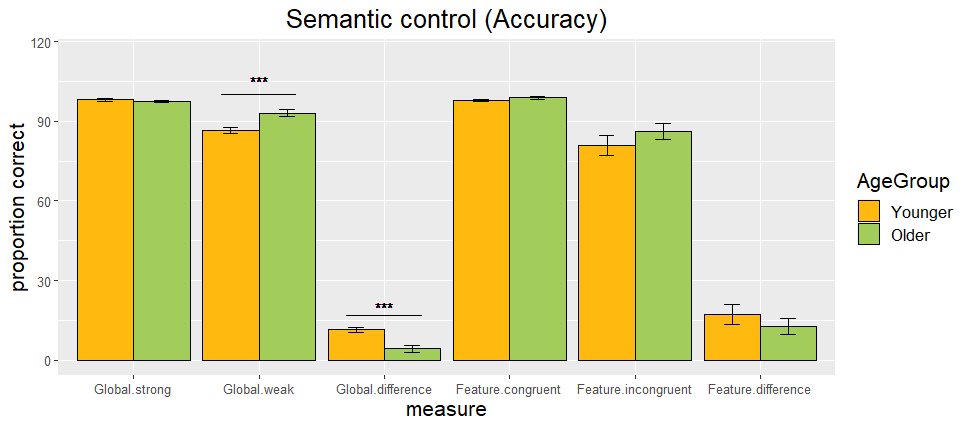
**

**
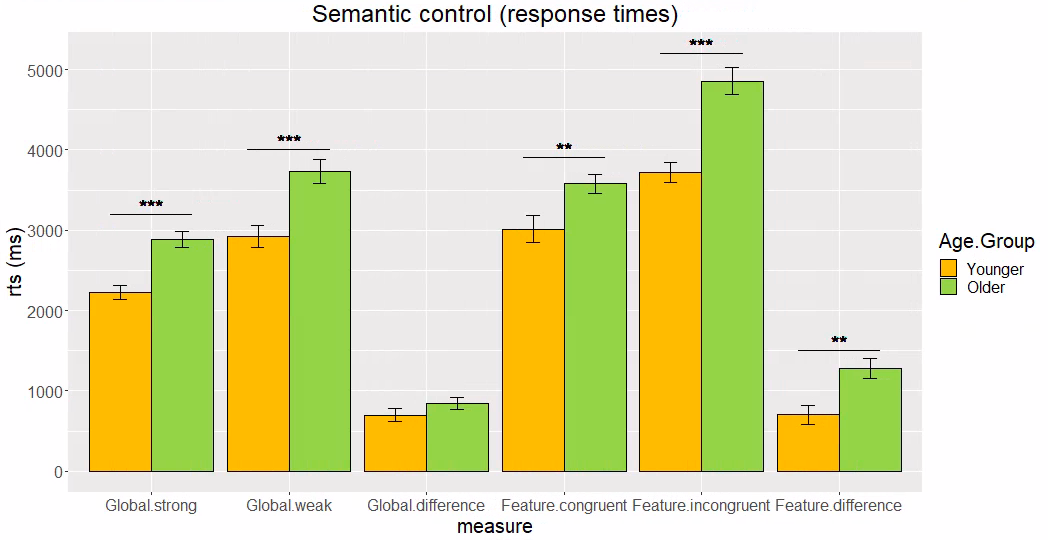
**

*Supplementary Figure 3.* Bar chart showing semantic control (accuracy, top; RTs, bottom) in the global association and feature association task. On the left, strong and weak global association trials are shown, with the global difference reflecting the difference between strong and weak trials. On the right, congruent and incongruent feature association trials are shown, with the feature difference reflecting the difference between congruent and incongruent trials.


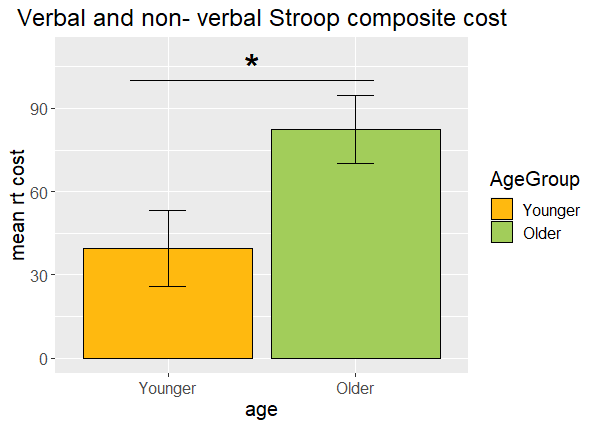


*Supplementary Figure 4.* Bar charts displaying mean Stroop interference costs (across the verbal and non-verbal tasks) per age group.

In the regression, as pre-registered, we created one Stroop cost across the verbal and non-verbal task. However, further checks showed the age-group difference was only significant in the verbal Stroop task (*M cost* older adults = 99ms, *SD* = 137; *M cost* younger adults = 39ms, *SD* = 104; t(80) = 2.216, p = .030), with no significant difference in the non-verbal task (*M cost* older adults = 66ms, *SD* = 66; *M cost* younger adults = 40ms, *SD* = 128; t(81) = 1.173, p = .244)


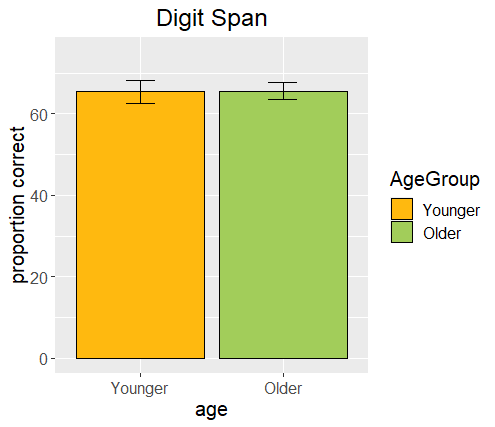


*Supplementary Figure 5.*  Bar charts displaying performance (percentage correct) on the digit span task per age group.

**Scatterplots**

The three plots below show the relationships between the cognitive tasks included in the regression analyses in Study 2 and the Match, Mismatch, and Context effects. All plots (across the three figures) use the same axis scales.


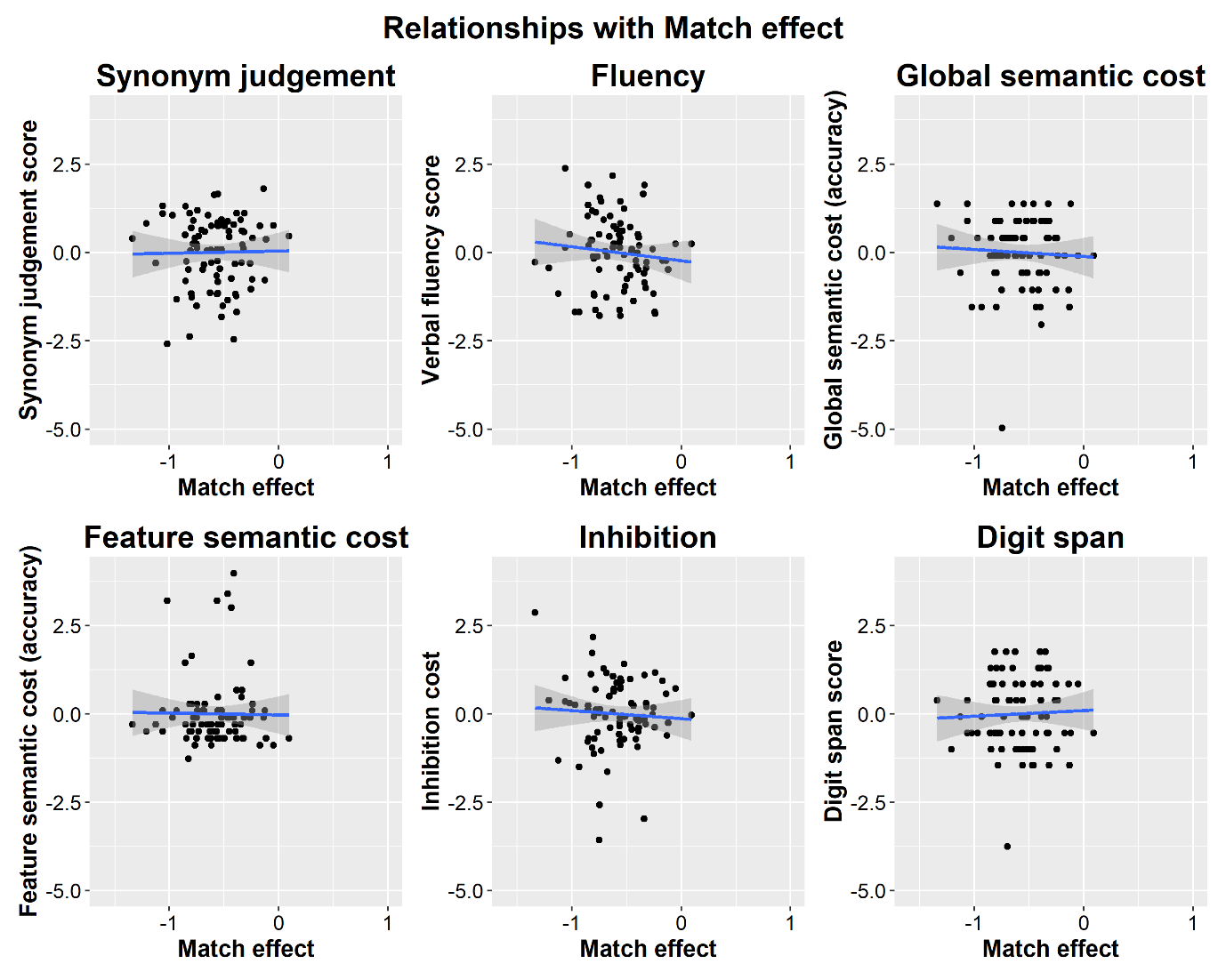


*Supplementary Figure 6.*  Scatterplots showing the relationships between the Match effect and the synonym judgement score (semantic knowledge), verbal fluency score, global semantic cost (accuracy), feature semantic cost (accuracy), inhibition cost, and digit span score.


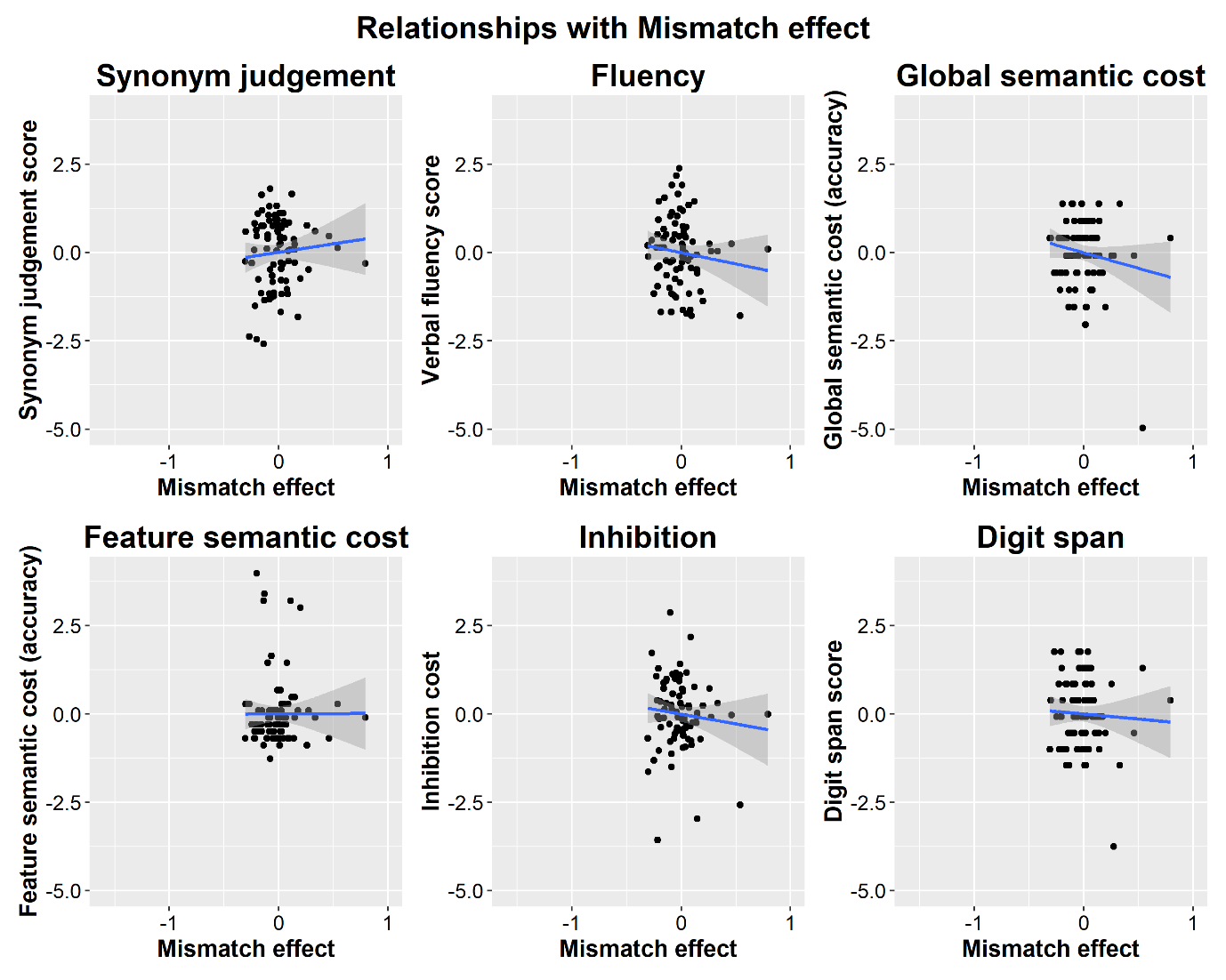


*Supplementary Figure 7.*  Scatterplots showing the relationships between the Mismatch effect and the synonym judgement score (semantic knowledge), verbal fluency score, global semantic cost (accuracy), feature semantic cost (accuracy), inhibition cost, and digit span score.

**
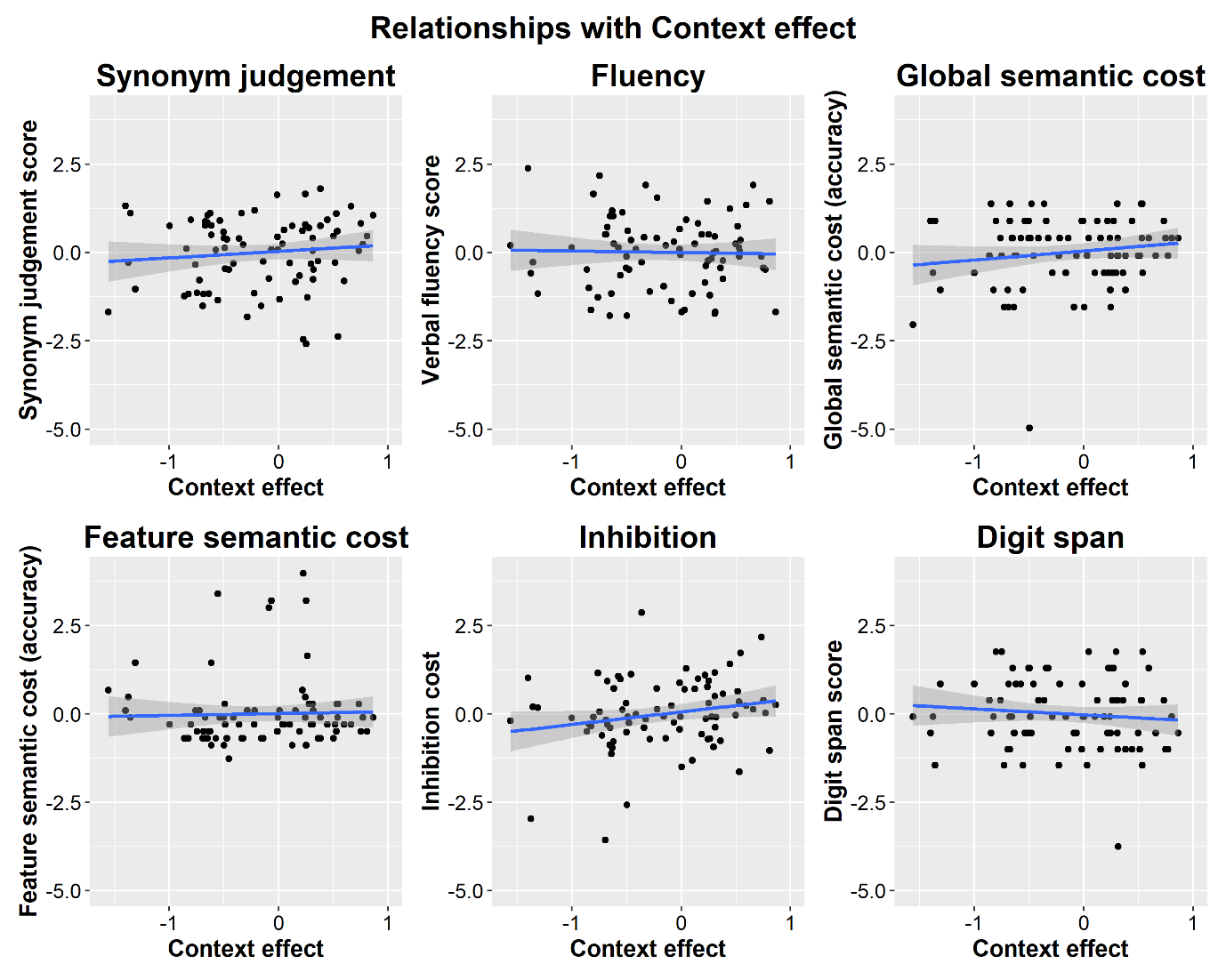
**

*Supplementary Figure 8.*  Scatterplots showing the relationships between the Context effect and the synonym judgement score (semantic knowledge), verbal fluency score, global semantic cost (accuracy), feature semantic cost (accuracy), inhibition cost, and digit span score.
